# Enhance the performance of current scoring functions with the aid of 3D protein-ligand interaction fingerprints

**DOI:** 10.1186/s12859-017-1750-5

**Published:** 2017-07-18

**Authors:** Jie Liu, Minyi Su, Zhihai Liu, Jie Li, Yan Li, Renxiao Wang

**Affiliations:** 1 State Key Laboratory of Bioorganic and Natural Products Chemistry, Collaborative Innovation Center of Chemistry for Life Sciences, Shanghai Institute of Organic Chemistry, Chinese Academy of Sciences, 345 Lingling Road, Shanghai, 200032 China; 2 State Key Laboratory of Quality Research in Chinese Medicine, Macau Institute for Applied Research in Medicine and Health, Macau University of Science and Technology, Macau, People’s Republic of China

**Keywords:** Protein-ligand binding affinity, Scoring function, Interaction fingerprints, Structure-based drug design

## Abstract

**Background:**

In structure-based drug design, binding affinity prediction remains as a challenging goal for current scoring functions. Development of target-biased scoring functions provides a new possibility for tackling this problem, but this approach is also associated with certain technical difficulties. We previously reported the Knowledge-Guided Scoring (KGS) method as an alternative approach (*BMC Bioinformatics*, 2010, 11, 193–208). The key idea is to compute the binding affinity of a given protein-ligand complex based on the known binding data of an appropriate reference complex, so the error in binding affinity prediction can be reduced effectively.

**Results:**

In this study, we have developed an upgraded version, i.e. KGS2, by employing 3D protein-ligand interaction fingerprints in reference selection. KGS2 was evaluated in combination with four scoring functions (X-Score, ChemPLP, ASP, and GoldScore) on five drug targets (HIV-1 protease, carbonic anhydrase 2, beta-secretase 1, beta-trypsin, and checkpoint kinase 1). In the in situ scoring test, considerable improvements were observed in most cases after application of KGS2. Besides, the performance of KGS2 was always better than KGS in all cases. In the more challenging molecular docking test, application of KGS2 also led to improved structure-activity relationship in some cases.

**Conclusions:**

KGS2 can be applied as a convenient “add-on” to current scoring functions without the need to re-engineer them, and its application is not limited to certain target proteins as customized scoring functions. As an interpolation method, its accuracy in principle can be improved further with the increasing knowledge of protein-ligand complex structures and binding affinity data. We expect that KGS2 will become a practical tool for enhancing the performance of current scoring functions in binding affinity prediction. The KGS2 software is available upon contacting the authors.

**Electronic supplementary material:**

The online version of this article (doi:10.1186/s12859-017-1750-5) contains supplementary material, which is available to authorized users.

## Background

Molecular docking has been an extremely powerful technique in structure-based drug design since the 1980s [1–4]. The primary goal of molecular docking is to predict the binding pose of a given ligand molecule to a molecular target, usually a protein or a nucleic acid. It provides a useful guide especially when experimental means, such as X-ray crystal diffraction or NMR spectroscopy, cannot supply the desired answer in a timely manner. To achieve this goal, molecular docking methods sample possible binding poses of the ligand molecule and often rely on a group of computational models called scoring functions [5–9] to rank them to select the preferred one. Based on the knowledge of the ligand binding pose, scoring functions are also employed to predict ligand binding affinity. As a useful expansion, large compound libraries can be screened computationally by using molecular docking methods to identify promising candidates that fit to a given target. Such “virtual screening” approaches are adopted nowadays by researchers in academia as well as pharmaceutical industry [10–12].

A number of evaluations of current docking/scoring methods [13–20] have suggested that they can provide reasonable predictions of ligand binding modes, but their performance is often disappointing in predicting ligand binding affinities. It is not totally surprising because protein-ligand binding is associated with sophisticated energetic factors. Accurate prediction of binding free energy remains as a major challenge even for high-level computational methods [21, 22]. If the scoring functions used in molecular docking could be improved in this aspect, molecular docking will certainly become more useful.

Most scoring functions are developed as all-purpose models, which are presumably applicable to all types of target protein. However, it is well-known that their performance varies significantly on different target proteins. Development of target-biased scoring function (or customized scoring function) has been proposed as a possible approach for improving the performance of current scoring functions [23]. A number of studies along this path have been reported in recent years. The most straightforward way to obtain a customized scoring function is to re-calibrate an all-purpose scoring function on a specific class of protein-ligand complexes [24–27]. For example, Pfeffer et al. developed DrugScore-RNA [24], which shares the same theoretical framework as DrugScore [28] but was derived from 670 nucleic acid-ligand and nucleic acid-protein complex structures. Antes et al. applied a parameter optimization method called POEM to re-calibrate two scoring functions (FlexX and ScreenScore) on complexes formed by kinases and ATPases [25]. Xue et al. developed the Kinase-PMF scoring function for evaluating the binding of ATP-competitive kinase inhibitors with a large set of kinase complexes [27]. Other methods for obtaining a customized scoring function (or scoring scheme) have also been reported. For example, Teramoto et al. reported supervised scoring models through feature selection to improve enrichment factors in virtual screening [29–31]. Avram et al. described a consensus scoring scheme, namely PLSDA-DOCET, which is geared towards five target proteins [32]. Their scoring scheme combines energy terms retrieved from several scoring functions in the FRED software, which produced promising results in virtual screening trials on an external test set [33].

In spite of the appealing prospects provided by customized scoring functions, they are associated with certain technical inconvenience in practice. An obvious limitation is that a new customized scoring function is needed whenever a new target protein is under consideration. It has been estimated that the human genome contains several thousands of druggable targets, which can be classified into at least several dozens of categories. It will need great efforts to develop customized scoring functions to tackle each of them. Moreover, re-calibration of an existing scoring function or formulation of a new model needs some special expertise, which is beyond the capability of most common end users. That is perhaps why customized scoring functions are not widely available yet.

**Fig. 1:**
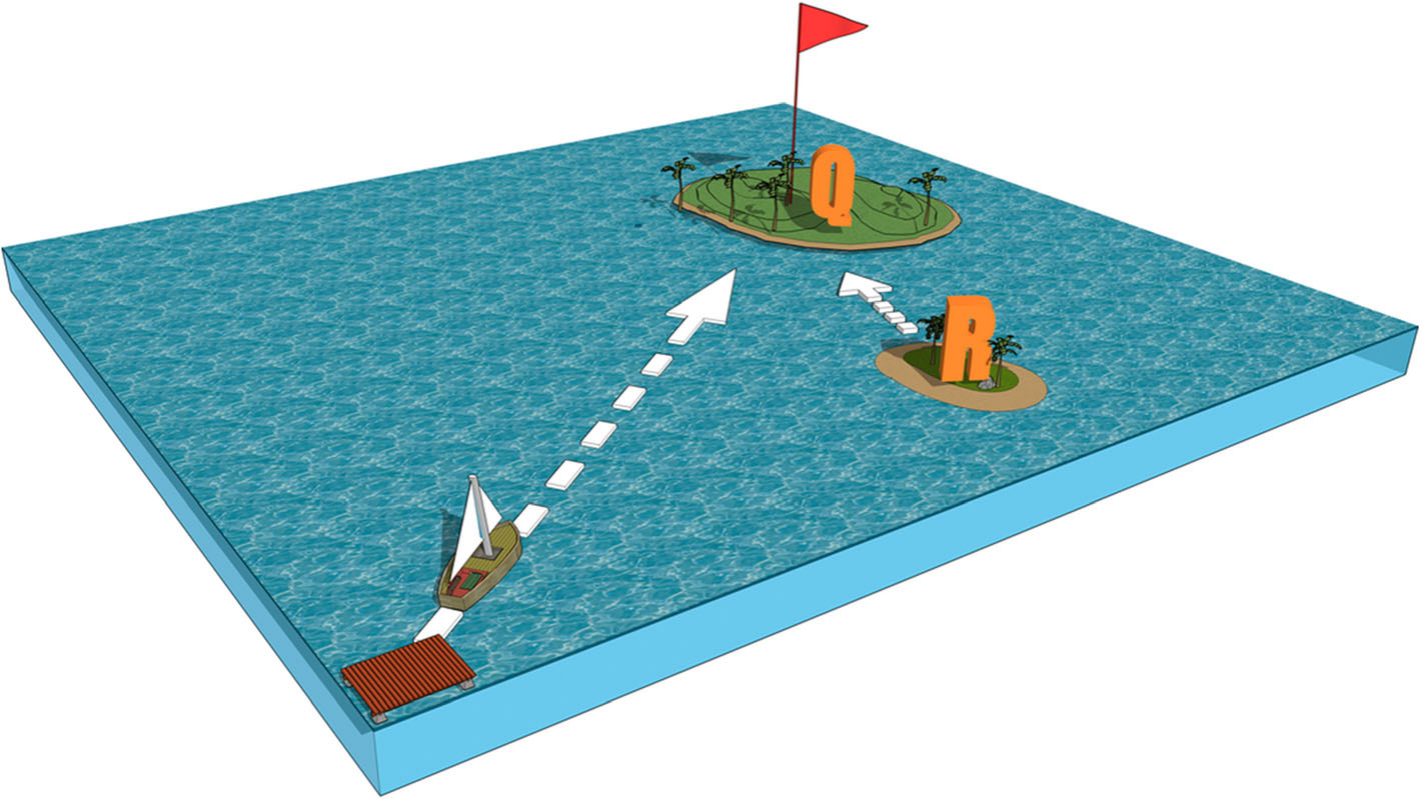
Illustration of the basic idea of the Knowledge-Guided Scoring (KGS) method. The sea represents the hypothetical “protein-ligand interaction space”. A given query complex (*Q*) is a small island somewhere in the sea. Binding affinity prediction by current scoring functions, most of which are additive models, is to sail from the origin of this space (at the lower-left corner) to the destination (*Q*). By the KGS method, if a reference complex (*R*) resembling the query complex can be found first, one can sail from the *R* island to the *Q* island for instead, which is assumed to be a less difficult journey

We have been seeking an alternative solution for common end users to enhance the performance of current scoring functions in binding affinity prediction without getting into the trouble of formulating customized scoring functions. Our solution is what we call the Knowledge-Guided Scoring (KGS) method. A prototype of this method was published previously in this journal [34]. Briefly, to compute the binding affinity of a query protein-ligand complex, an appropriate reference complex with known binding data needs to be defined first (see Fig. 1 for a conceptual illustration), which is required to resemble the query complex. Then, a standard scoring function is used to compute both the query and the reference. The binding score computed for the query is adjusted with the known binding data of the reference. In this way, certain structural or energetic factors on these two complexes may cancel out, so the final adjusted binding score is expected be closer to the true value. We demonstrated that application of KGS indeed produced more accurate binding scores than scoring functions alone on several target proteins [34]. In the technical aspect, KGS can be applied in combination with any scoring function, and no re-engineering on the partner scoring function is needed. Thus, it represents a more flexible option in practice than customized scoring functions.

As a notable new trend in the field of structure-based drug design, structural interaction fingerprints have emerged as a new approach for evaluating protein-ligand interactions [35]. An pioneering work was conducted by Deng et al. [36]. The key idea was to encode the 3D structural information of a protein-ligand complex into a 1D binary string (i.e. the fingerprints) recording the typical interactions between the ligand molecule and a set of pocket residues. Later, such fingerprints were extended in various ways to encode more specific information of protein-ligand interactions at the atomic level [37–44]. More recently, some researchers developed interaction fingerprints in 3D forms, which were based on ligand binding modes, target protein structures, or protein-ligand complex structures [45–50]. A major application of those interaction fingerprints is to re-rank ligand docking poses based on their similarity to the known binding modes of relevant reference molecules. Indeed, interaction fingerprints often outperformed standard scoring functions in terms of identifying correct ligand binding modes and recovering active compounds in virtual screening trials conducted on a range of target proteins. Moreover, interaction fingerprints are also used to compare protein binding pockets, evaluate the structural diversity of the ligands generated by automated methods, and so on.

Inspired by the concept of protein-ligand interaction fingerprints, we have re-composed the algorithm used by KGS in reference selection. The new implementation will be referred to as KGS2 in this article. In the original KGS method, the reference complex is selected by comparing target-based pharmacophore features deduced inside the binding pocket; whereas in KGS2, the reference complex is selected by comparing 3D protein-ligand interaction fingerprints. We have tested KGS2 in combination with four popular scoring functions. In situ scoring tests were conducted on experimental complex structures formed by five target proteins. Application of KGS2 indeed produced more accurate binding scores than scoring functions alone in most cases. Besides, KGS2 always outperformed the original KGS method. Molecular docking tests were conducted on four additional data sets, each of which consisted of some congeneric ligand molecules for one target protein. Application of KGS2 also led to somewhat improved results. We demonstrate in this study that the performance of current scoring functions in binding affinity prediction can be enhanced by KGS2 with the aid of 3D protein-ligand interaction fingerprints.

## Methods

### The overall strategy

Our KGS2 method follows the same approach as the original KGS [34]. The binding affinity of a query protein-ligand complex (*Q*) is computed by a scoring function (*SF*) as:

1 $$ {\widehat{Q}}_{bind}= b+ k\times {Q}_{score, SF} $$

Here, *Q*_*score* , *SF*_ is the binding score of *Q* computed by *SF*. Introduction of parameter *k* and *b* is necessary for correlating the binding scores computed by *SF* to experimental binding data because binding scores are often in an arbitrary unit or their values may not be in a range comparable to experimental binding data. Similarly, the binding affinity of an appropriate reference complex (*R*) computed by *SF* is:

2 $$ {\widehat{R}}_{bind}= b+ k\times {R}_{score, SF} $$

By subtracting Eq. 2 from Eq. 1, one has:

3 $$ {\widehat{Q}}_{bind}={\widehat{R}}_{bind}+ k\times \left({Q}_{score, SF}-{R}_{score, SF}\right) $$

Replacing the predicted binding affinity of the reference complex (${\widehat{R}}_{bind}$) in Eq. 3 with its known experimental value (*R*_*exp*_), one gets:

4 $$ {\widehat{Q}}_{bind}={R}_{exp}+ k\times \left({Q}_{score, SF}-{R}_{score, SF}\right) $$

Equation 4 indicates how KGS2 computes the binding affinity of a given protein-ligand complex using the known binding affinity of a reference complex. Here, *k* is an adjustable parameter associated with scoring function *SF*. This parameter can be derived through a standard linear regression between the binding scores computed by *SF* and the experimental binding data of a set of protein-ligand complexes, where the slope of the regression line gives this parameter. In this study, the PDBbind “refined set” (version 2014) was employed as the training set to derive the required *k* parameter for each scoring function. This data set consists of 3446 protein-ligand complexes with known 3D structures and binding constants, which are selected by a set of quality control filters from the entire PDBbind database [16].

By KGS2, the reference complex for a given query complex is determined by searching among a reference library, i.e. an external data set of protein-ligand complexes with known 3D structures and binding data. The complex in this library sharing the highest 3D similarity to the query complex will be selected as the reference and used in Eq. 4. During this process, each complex structure is analyzed to derive a set of 3D protein-ligand interaction fingerprints. A number of dispersed “interaction patterns” are elucidated from the interaction fingerprints, which are intended to cover the key factors in protein-ligand interaction. The similarity between any two complexes is then assessed by detecting the maximal mapping between their interaction patterns. The algorithms used in this process are explained in the following sections.

### Extraction of protein-ligand interaction units

**Fig. 2:**
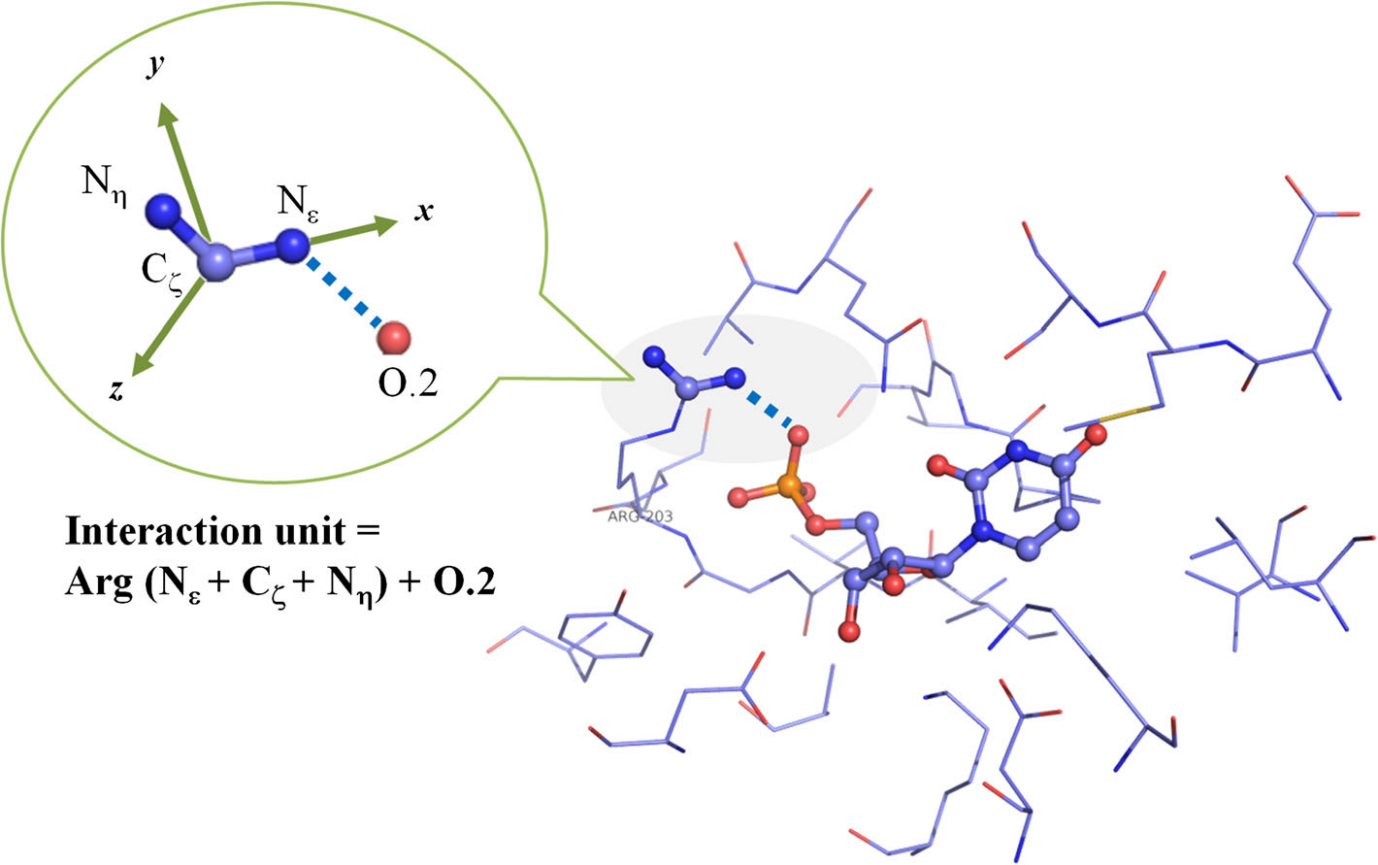
Illustration of an interaction unit between the side chain of an Arg residue and a phosphate group on the ligand molecule (PDB entry 1LOQ)

The basic elements in our 3D fingerprints are “interaction units”. An interaction unit is composed of four atoms, including three covalently linked atoms on the protein molecule and one atom on the ligand molecule (Fig. 2). Our concept of interaction unit was inspired by the work by Kinoshita et al. [51], who analyzed a larger number of protein-ligand complex structures to derive the spatial distribution of ligand atoms around fragments on protein molecules. In each interaction unit, the distance between the ligand atom and the nearest protein atom should be shorter than the sum of their van der Waals radii plus a margin of 1 Å. This is to ensure that each interaction unit under consideration is involved in direct protein-ligand contact. Each interaction unit is represented by a string including the standard PDB names of the three protein atoms plus the residue name (e.g. “Asp: O − C − C_α_”) and the SYBYL Mol2 atom type of the ligand atom (e.g. “O.2”). For the sake of convenience, the three atoms on the protein side in each interaction unit will be referred to as the “protein fragment” in this article. An interaction unit is characterized by its components as well as geometry. Geometry of an interaction unit is represented by the relative coordinates of the ligand atom in a local Cartesian coordinate system defined by the protein fragment. In this coordinate system, the origin locates at the protein atom in the middle, the *xy* plane is defined by the three protein atoms, and the direction of the *z* axis points toward the same side as the ligand atom (Fig. 2).

The PDBbind “general set” (version 2014) [52], which provides the experimental binding data as well as processed structural files of 10,605 diverse protein-ligand complexes in PDB, was employed to extract the interaction units observed on protein-ligand binding interfaces. The contacting atom pairs between the protein and the ligand in each complex structure were examined, and then all possible interaction units containing these contacting atom pairs were recorded. A total of 6,762,383 interaction units were extracted from those complex structures. In terms of components, these interaction units belonged to 9570 different types.

### Detection of protein-ligand interaction patterns

In our method, “interaction patterns” refer to the interaction units with a higher level of statistical preference, which are presumed to be the key factors in protein-ligand interaction. In order to detect such interaction patterns, the interaction units recorded at the previous step were analyzed. First, if a certain type of interaction unit had an occurrence below 100, it was ignored due to lack of significance. Then, the geometry of each remaining type of interaction unit was examined by using an algorithm based on the Gaussian Mixture Model (GMM) [53]. The same algorithm was employed by Rantannen et al. to investigate the spatial distributions of protein atoms around some pre-defined ligand fragments [54] as well as in Kinoshita’s study [51]. A probability density function p(*x*) was used to describe the event when a ligand atom at position *x* in the local coordinate system interacts with a protein fragment:

5 $$ \mathrm{p}(x)=\sum_{k=1}^K{\pi}_k N\left( x|{\mu}_k,{\Sigma}_k\right) $$

Here, p(*x*) is computed as the sum of a number of Gaussian components. $N\left( x|{\mu}_k,{\Sigma}_k\right)$ is a Gaussian distribution with a peak at *μ*_*k*_ and a covariance matrix of ${\Sigma}_k$. *π*_*k*_ is a weight factor for this Gaussian component. The parameters ${\mu}_k{,\Sigma}_k$ and *π*_*k*_ were all derived by maximizing the likelihood of the data point *x* in the distribution given by GMM through a variational Bayesian analysis. The maximal number of Gaussian components in each GMM, i.e. *K*, was set to 15 by default. Then, *K* was reduced during a learning process where parameter *π*_*k*_ was adjusted to zero for unnecessary Gaussian components. Then, each remaining Gaussian component, if it had an occurrence over 100 and its weight factor *π*_*k*_ ≥ 0.01, was recorded as a significant interaction pattern.

In plain words, the above process derived the preferred positions of the ligand atom relative to the protein fragment in each type of interaction unit. Each of them represents a preferred geometry of this type of interaction unit. For the 9570 different types of interaction units recorded at the previous step, a total of 16,272 interaction patterns were detected.

**Fig. 3:**
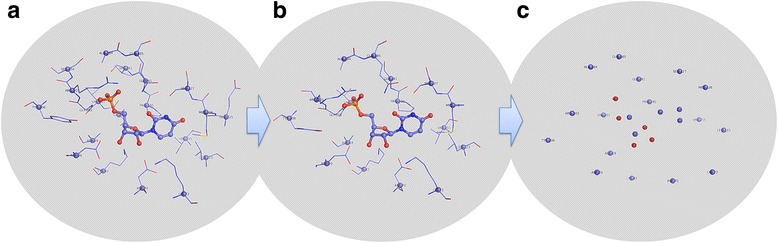
Illustration of how the 3D interaction fingerprints used in structural comparison are generated. **a** The original binding pocket and the ligand molecule. **b** Only the pocket residues carrying an interaction pattern are kept. **c** Each interaction pattern is then degraded into a pair of nodes, where one node is placed on the alpha-carbon of the residue and the other on the ligand atom relevant to this interaction pattern

Then, the key protein-ligand interactions in a given complex structure can be represented by a set of interaction patterns (Fig. 3). For this purpose, the ligand binding pocket on the target protein was defined first to include all amino acid residues within 4.5 Å from the ligand molecule. Next, all interaction units formed between pocket residues and the ligand molecule were extracted (Fig. 3a). Each interaction unit was examined to see if it matched to any of the 16,272 recorded interaction patterns. The Mahalanobis distance [55] between a given interaction unit (*x*) and a Gaussian component of an interaction pattern of the same type (*g*) was computed as [53]:

6 $$ D\left( x, g\right)=\sqrt{{\left( x-{\mu}_g\right)}^{\mathrm{T}}{\Sigma}_g^{-1}\left( x-{\mu}_g\right)} $$

If *D*(*x*, *g*) was smaller than 2.5, it was considered as a match between *x* and *g*. Here, an interaction unit *x* could be assigned to more than one Gaussian component. This process was repeated until all extracted interaction units had been examined. The outcome was a complete set of protein-ligand interaction patterns observed in the given complex structure (Fig. 3b).

Here, a technical issue is that interaction patterns cannot be used directly to compare two complex structures. It is because each complex is typically composed of more than a dozen of interaction patterns (each of which has four atoms), which is too sophisticated for designing an efficient mapping algorithm based on atomic coordinates. Some simplifications are thus necessary here. In our method, an interaction pattern is degraded into a pair of nodes: One node locates on the ligand atom, which records the type of the ligand atom (e.g. “O.2”); while the other locates on the backbone C_α_ atom of the residue containing the protein fragment, which records the type of the residue (e.g. “Arg”). In this way, the complete set of interaction patterns is now simplified into a set of nodes in space (Fig. 3c). The pocket residues that do not contribute any interaction pattern are not included in this set of nodes.

### Selection of the reference complex

**Fig. 4:**
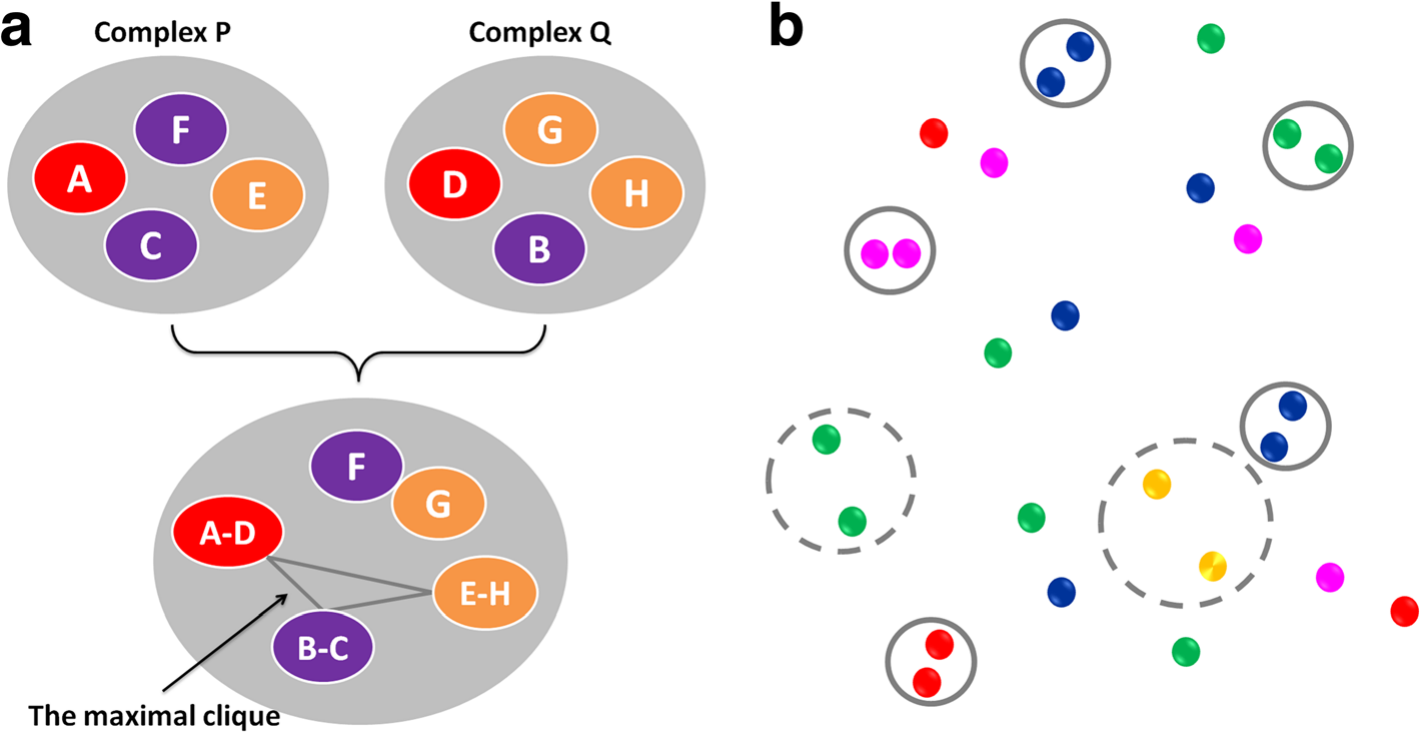
How the interaction fingerprints of two complexes (*P* and *Q*) are compared. **a** First, the maximal clique between node sets *P* and *Q* is defined. Each element in this maximal clique is a matched pair of nodes. **b** Then, the matched node pairs in the maximal clique (*in solid or dashed circles*) are superimposed. If the two nodes in a matched pair are close enough (d < 1 Å), they are considered as geometrically overlapped (those in *solid circles*). Overlapped node pairs are used in the computation of the similarity index between *P* and *Q*

By KGS2, the best reference complex for a query complex is the one in the reference library sharing a maximal subset of interaction patterns with the query complex. As mentioned above, each interaction pattern can be simplified into a single node. Our algorithm for finding the maximal common subset between two sets of nodes is illustrated in Fig. 4 with a simplified example. At the first step, all matched pairs of nodes between two sets (*P* and *Q*) are detected. Here, two matched nodes must have the same residue type or ligand atom type. A hypothetical graph *G* is generated using each matched pair of nodes as a new node. Two nodes, e.g. *A-D* and *B-C*, are connected with an edge if the *A-C* distance in set *P* is close enough to the *B-D* distance in set *Q* (Fig. 4a). Two distances, e.g. *d*_1_ and *d*_2_, are considered to be close enough if *d*_1_ < *k*·*d*_2_ (when *d*_1_ > *d*_2_) or *d*_2_ < *k*·*d*_1_ (when *d*_1_ < *d*_2_), where *k* is an adjustable parameter with a default value of 1.1. Then, the Born-Kerbosch algorithm for clique detection [56] is applied to identify the maximal clique in graph *G*. At the second step, sets *P* and *Q* are superimposed by considering only the nodes in the maximal clique. Then, a matched node pair is considered to be geometrically “overlapped” if the distance between them is shorter than 1 Å. Among all possible solutions of superimposition, only the one with the maximal number of overlapped node pairs is retained (Fig. 4b).

In KGS2, a minimum of five pairs of overlapped nodes are required to define complex *P* as a possible reference complex for the query complex *Q*. Above this threshold, the similarity index (*SI*) between *P* and *Q* is calculated by the classical Tanimoto coefficient [57]:

7 $$ {SI}_{p q}=\frac{N_{p q}}{N_p+{N}_q-{N}_{p q}} $$

Here, *N*_*p*_ and *N*_*q*_ are the numbers of nodes in the interaction fingerprints of *P* and *Q*, respectively; *N*_pq_ is the maximal number of overlapped nodes between *P* and *Q*. In order to search for the reference complex for a query complex, each complex in the chosen reference library is analyzed with the algorithms described through section “Extraction of protein-ligand interaction units” to “Detection of protein-ligand interaction patterns”, and its similarity to the query complex is assessed using Eq. 7. Here, one can also set a minimal similarity index required in reference selection, i.e. the similarity index between each candidate reference complex and the query complex must be higher than this cutoff value. Then, the final reference complex is selected as the one sharing the highest similarity index to the query complex.

### Preparation of the reference library

The reference library used by KGS2 is an assembly of known protein-ligand complex structures. Importantly, the experimental binding data of each complex should be available, which will provide the reference binding data (*R*_exp_) required in Eq. 4. In this study, the PDBbind “general set” (version 2014) [52] was employed by us as the default reference library in all test cases. This data set includes 10,605 complexes formed between diverse proteins and small-molecule ligands, each of which has known 3D structure from PDB and experimental binding data (*K*_d_, *K*_i_, or *IC*_50_) curated from literature. This data set is the largest one of this type in public domain and thus is a good choice for our purpose. Each complex structure was processed using methods described in our previous work [16, 17], where the protein molecule was saved in a PDB format file and the ligand molecule was saved in a Mol2 or SDF format file. KGS2 read in each complex structure, analyzed all of the protein-ligand interaction units, and then output the selected interaction patterns into a special data file. It took KGS2 roughly 8 s to analyze one complex structure and retrieve the interaction patterns by a single-CPU job. It took a whole day to process the entire PDBbind general set (10,605 complexes). Nevertheless, this process needs to be conducted only once for a chosen library, and thus it is not a problem at all.

In fact, the computation time needed by KGS2 is consumed mainly on comparing the given query complex with each complex in the reference library. The computation time needed for this job is roughly proportional to the binding interface on the query complex. At average, it took KGS2 around 6 min to screen the pre-processed PDBbind general set (i.e. ~30 complexes per second) by a single-CPU job. Note that this process can be easily accelerated through parallel jobs. Moreover, in reality one probably will not use a comprehensive, non-discriminatory reference library as the PDBbind general set. A more practical approach is to use a smaller, focused reference library, which is composed of, for example, complexes formed by the same protein molecule as the query complex. Application of KGS2 in that way will not require a significant amount of computation time. Thus, KGS2 can work with fast scoring functions nicely.

The computation time of KGS2 reported above was obtained by conducting a single-CPU job in a “clean” environment on a Dell Precision T5610 desktop workstation (dual Intel Xeon E5–2609 v2 CPU @ 2.50GHz, Intel C602 chipset, 16 GB DDR3 memory) running the 64-bit RedHat 6.4 Linux operation system.

### Variations of the standard model

The standard model of KGS2 is described through section “The overall strategy”–“Selection of the reference complex” above. In order to make a comparison, three variations were also considered in our study. As the standard model, these variations all relied on Eq. 4 to compute the binding affinity of a query complex.

Variation Model 1: This variation differed from the standard model in how the adjustable parameter *k* in Eq. 4 was derived. In the standard model, the parameter *k* for each scoring function under consideration was derived through a regression analysis on the entire PDBbind refined set (3446 complexes in total). Note that there were overlaps between the refined set and the five data sets used in our in situ scoring test. In order to investigate if such overlapping complexes could introduce bias into the final results produced by KGS2, all *k* parameters used in this variation model were derived on the remaining 2859 complexes in the refined set after excluding the complexes overlapping with the five test sets. All other aspects of this variation model were the same as the standard model.

Variation Model 2: This variation differed from the standard model in the algorithm used for reference selection. It was designed to investigate if the 3D interaction fingerprints used in KGS2 was indeed superior to an algorithm that did not rely on 3D structural information. To compute a given query complex with this variation, the first step was to detect among the entire reference library the complexes formed by the same protein as the query complex. For this purpose, the query complex was compared to each complex in the reference library in terms of protein sequence similarity. If the similarity was above 95%, the two complexes were considered to be formed by the same protein. Here, The similarity between two protein sequences was computed with the CD-hit software released by PDB [58]. At the second step, 2D structure of the ligand in the query complex was compared to the ligands in those complexes detected at the previous step. The similarity between two ligands was computed with the ECFP fingerprints by using the CANVAS module in the Schrödinger software (version 9.3.5, Schrödinger Inc.). The final selected reference complex was the one that shared the highest 2D ligand similarity with the query complex.

Variation Model 3: This variation also differed from the standard model in the algorithm used for reference selection. It was designed to investigate if the 3D interaction fingerprints used in KGS2 was superior to an algorithm that was based only on the 3D protein structural information. With this variation, comparison of two complex structures also utilized the interactions patterns identified between the protein and the ligand (Fig. 3c). However, only the nodes associated with pocket residues were considered in comparison; while the nodes associated with ligand atoms were ignored. All other aspects of this variation model were the same as the standard model.

### The first type of test: In situ scoring

KGS2 was first validated in so-called “in situ scoring” test, where each scoring function was applied in combination with KGS2 to protein-ligand complexes with known 3D structure to compute their binding affinities. In addition, the three variation models as well as the original KGS method were also tested in order to make a comparison. Performance of each combined scoring scheme was assessed by the correlation between the computed binding scores and the experimental binding data of those complexes. Five well-established drug targets, including HIV-1 protease, carbonic anhydrase 2 (CA-2), beta-secretase 1 (BACE-1), beta-trypsin, and checkpoint kinase 1 (CHK-1), were selected as the test cases. All five proteins are established drug targets. A significant number of complexes formed by each of them are available, which is essential for achieving statistical significance in subsequent analysis. The complexes formed by these target proteins in the PDBbind general set (version 2014) were retrieved, including 304 HIV-1 protease complexes, 230 CA-2 complexes, 223 BACE-1 complexes, 196 trypsin complexes, and 61 CHK-1 complexes, respectively (see the Additional file 1: Table S1 and Figure S1). In addition to experimental binding data, processed structural files for all complexes (i.e. protein molecules in the PDB format and ligand molecules in the SYBYL Mol2 and SDF format) were also obtained from the PDBbind database. The methods for processing those complex structures have been described in our previous publication [17].

Four scoring functions were considered in this test, including three scoring functions implemented in the popular GOLD software (version 5.2, Cambridge Crystallographic Data Center), i.e. ChemPLP [59], ASP [60], and GoldScore [61], and a standalone scoring function X-Score (version 1.3) [62]. Among them, ChemPLP and X-Score are empirical scoring functions, ASP is based on knowledge-based statistical potentials, while GoldScore is essentially a force field-based model. Moreover, they are the relatively successful ones in each category according to the results obtained on some benchmarks [15, 17]. Technically, it is also convenient to apply these scoring functions because they all directly accept the processed structural files provided by PDBbind as inputs.

Then, all four scoring functions were applied to the five test sets. For each test set, the binding scores of all member complexes were computed first by applying those scoring functions alone. Next, the default reference library used by KGS2 (i.e. the PDBbind general set) was searched to select the reference complex for each complex in the test set. Because all five test sets under our consideration were also selected from the PDBbind general set, the reference complex selected in each case was examined to ensure that it was not identical to the query complex (otherwise one would obtain 100% accurate “predictions”). If a qualified reference complex was found, adjusted binding scores for all four scoring functions were computed with Eq. 4 based on the known binding data of the reference complex. If not, the binding scores were computed with Eq. 1. In either case, the computed binding scores were given as binding constants in logarithm (i.e. log*K*_a_). Finally, the Pearson correlation coefficient (*R*_p_) between the experimental binding data and the computed binding scores for the entire test set was calculated for each scoring function. The standard deviation (*SD*) in fitting the computed binding scores to the experimental binding data was used as a quantitative indicator of accuracy in subsequent analysis. *SD* was chosen instead of *R*_p_ for this purpose because *SD* is a quantity independent of sample size.

### The second type of test: molecular docking

Our second type of test attempted to reflect the reality in structure-based drug design more closely. The aim was to model the structure-activity relationship of a congeneric set of ligand molecules through molecular docking and scoring. To select the appropriate test sets, we focused on the target proteins already considered in the in situ scoring test. One data set for HIV-1 protease, CA-2, BACE-I, and CHK-1, respectively, were selected among the “validation sets” from BindingDB (http://www.bindingdb.org/validation_sets/) [63]. Trypsin was excluded here because there was no validation set of trypsin inhibitors in the current release of BindingDB (as by April, 2016). In order to select the data sets employed in our study, each data set must contain at least 10 ligand molecules with experimental binding data, and the binding affinity range must be larger than 10 folds. Besides, each data set was required to be retrieved from a relatively recent study (e.g. published in the last 10 years). The basic information of the four selected data sets is summarized in Table 1.

**Table 1:** Basic information of the four test sets used in the molecular docking test

Target protein	Number of ligands	Binding affinity range (nM)	PDB ID of the template complex structure	References given by BindingDB
HIV-1 protease	12	0.0045–5.3	2HB3	*J Med Chem*, 2006, 49:5252–61; *J Med Chem*, 2009, 52:7689–705
Carbonic anhydrase 2	15	20–330	3MYQ	*Bioorg Med Chem*, 2010, 18:7357–64; *Eur J Med Chem*, 2012, 51:259–70.
Beta-secretase 1	14	3–380	2VJ7	*Bioorg Med Chem Lett*, 2008, 18:1022–6; *Bioorg Med Chem Lett*, 2009, 19:3664–8
Checkpoint kinase 1	15	5–50,000	3U9N	*ACS Med Chem Lett*, 2012, 3:123–128.

As a useful feature of the validation sets from BindingDB, the crystal complex structure of at least one ligand molecule in each data set is available from PDB. In our study, this particular complex structure was used as the template for deriving the binding modes of all ligand molecules in the same data set. For each ligand molecule, the GOLD software (version 5.2, Cambridge Crystallographic Data Center) was employed to generate up to 100 ligand binding poses. The protein structure was kept fixed during this process. The binding pocket was defined by using the native ligand molecule in the crystal complex structure with an envelop of 10 Å. The “200% searching efficiency” parameter set was applied during the sampling process, where the ChemPLP scoring function in GOLD was chosen for ranking the generated ligand binding poses. In order to obtain results in consistence with the other ligands in the same data set, binding poses of the ligand in the template complex structure were also generated through the same procedure.

The same four scoring functions (ChemPLP, ASP, GoldScore, and X-Score) were tested in combination with KGS2 in this test. To predict the binding affinity of a ligand molecule, each scoring function was applied alone first to rank all binding poses of this ligand by binding scores computed with Eq. 1. The binding score of the top-ranked binding pose was recorded as the binding affinity predicted by this scoring function. Next, this scoring function was applied in combination with KGS2 to re-rank all ligand binding poses by the adjusted binding scores computed with Eq. 4. Here, the reference library used by KGS2 was also the PDBbind general set (version 2014). The similarity cutoff for selecting the reference complex was set to 0.10. This low cutoff was adopted in order to increase the chance of finding a reference. In case that a reference could not be found for the given complex, the binding score was computed with Eq. 1 for instead. After all binding poses were re-processed in this way, the binding score of the top-ranked binding pose was recorded as the binding affinity predicted by KGS2. After all ligand molecules in a test set were computed through the above process, the correlation between the experimental and the predicted binding data (including the original binding scores produced by each scoring function alone and the adjusted binding scores produced by applying KGS2) was analyzed. The one achieving a higher correlation with the experimental binding data was considered to be more accurate.

Our results obtained in the in situ scoring test indicated that the performance of the three variation models and the original KGS method was generally inferior to the standard model of KGS2 (see Performance of three variation models in the in situ scoring test). Thus, those models were not considered further in this test.

## Results and discussion

### KGS2 versus KGS

**Fig. 5:**
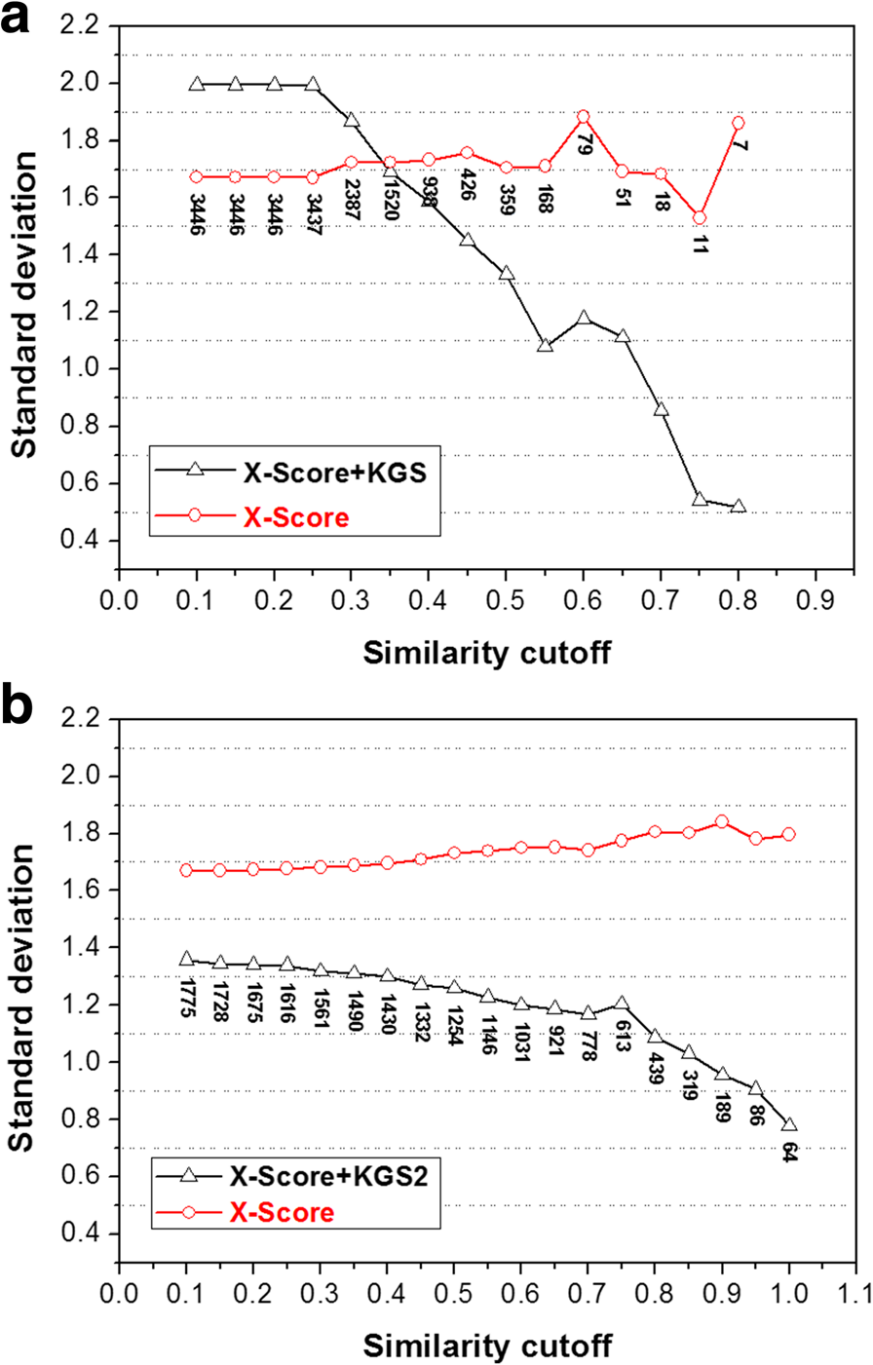
Comparison of the performance of KGS2 and KGS on the PDBbind refined set (version 2014). **a** The results given by X-Score + KGS; **b** The results given by X-Score + KGS2. In both figures, the *x-axis* indicates the similarity cutoff required in reference selection; The y-axis indicates the standard deviation (in log*K*_a_ units) in fitting the computed binding scores to the experimental binding data on a particular subset of complexes. The *number* near each data point indicates the size of each subset, i.e. the number of complexes for which a reference complex can be found at this level of similarity cutoff. Results produced by X-Score alone are indicated by *red round* data points. Results produced by X-Score + KGS or X-Score + KGS2 are indicated by *black triangular* data points. Application of KGS or KGS2 produces more accurate results than the scoring function alone when the *black line* is below the *red line*

KGS2 is developed as an upgrade of the original KGS method. Therefore, we compare the performance of KGS2 and KGS first. The results produced by the X-Score scoring function in combination with KGS2 and KGS on the entire PDBbind refined set are illustrated in Fig. 5. Here, the advantage of KGS2 over KGS can be seen in two aspects. Firstly, there is a “critical point” for X-Score + KGS to produce more accurate binding scores than X-Score alone, i.e. when the similarity cutoff required in reference selection is above 0.35 (Fig. 5a). This observation is consistent with what was observed on smaller data sets in our previous study [34]. In the case of KGS2, however, there is no such a critical point (Fig. 5b). The binding scores produced by X-Score + KGS2 are always more accurate in a statistical sense than X-Score alone as long as appropriate references are available. Even at the lowest similarity cutoff applied to reference selection (i.e. *SI* ≥ 0.10), the errors produced by X-Score + KGS2 are smaller by 0.3 log*K*_a_ units (corresponding to one-fold difference in binding constant) than those produced by X-Score alone. Moreover, X-Score + KGS2 achieves this level of improvement (i.e. smaller errors by 0.3 log*K*_a_ units) for nearly 1800 complexes in this data set. In contrast, X-Score + KGS achieves the same level of improvement for about 400 complexes. In this sense, KGS2 is about four times more effective than KGS on this data set.

Secondly, one would expect KGS2 to produce a more accurate prediction if the selected reference complex resembles the query complex more closely. Indeed, one can see that the advantage of X-Score + KGS2 over X-Score alone becomes more obvious where higher similarities are required in reference selection (Fig. 5b). When the required similarity is very high, e.g. *SI* ≥ 0.90, the errors produced by X-Score + KGS2 are smaller than X-Score alone by almost one log*K*_a_ unit (i.e. ten-fold in binding constant). The same trend is also observed for X-Score + KGS at higher levels of required similarity (Fig. 5a). However, the number of complexes to which KGS is applicable drops rapidly in such circumstances. For example, after the required similarity is above 0.70, KGS is applicable to less than two dozens of complexes; while KGS2 is still applicable to nearly 800 complexes.

These observations suggest that KGS2 is generally more effective and more robust than the original KGS method, which should be attributed to the new algorithm designed for reference selection. The original KGS method generates a target-based pharmacophore model inside binding pocket and then relies on it for selecting the reference complex. Although KGS indeed produced improved results in some test cases [34], we realized later that too much protein-ligand interaction information was actually lost during deduction of a target-based pharmacophore model. A pharmacophore model carries rather limited information because it consists of only a small number of features in several categories (e.g. hydrogen bond donor, hydrogen bond acceptor, positive/negative charge center, and hydrophobic core). Moreover, structural information at the ligand side is completely ignored by KGS. Therefore, we turned to 3D protein-ligand interaction fingerprints for instead to develop KGS2. In literature, protein-ligand interaction fingerprints can be generated with various algorithms, ranging from 1D, 2D to 3D descriptors [35–50]. Our 3D interaction fingerprints are based on the “interaction patterns” derived through a statistical analysis of a large set of protein-ligand complex structures. One set of interaction fingerprints usually contains a much larger number of elements (around 30 interaction patterns on average, no upper limit) than a target-based pharmacophore model used in KGS (around 8 features on average, up to 15). Besides, such interaction fingerprints combine 20 residue types and 25 ligand atom types, which carry more detailed information than a simple pharmacophore model. Thus, KGS2 is in theory a better method than KGS for encoding protein-ligand interactions.

**Fig. 6:**
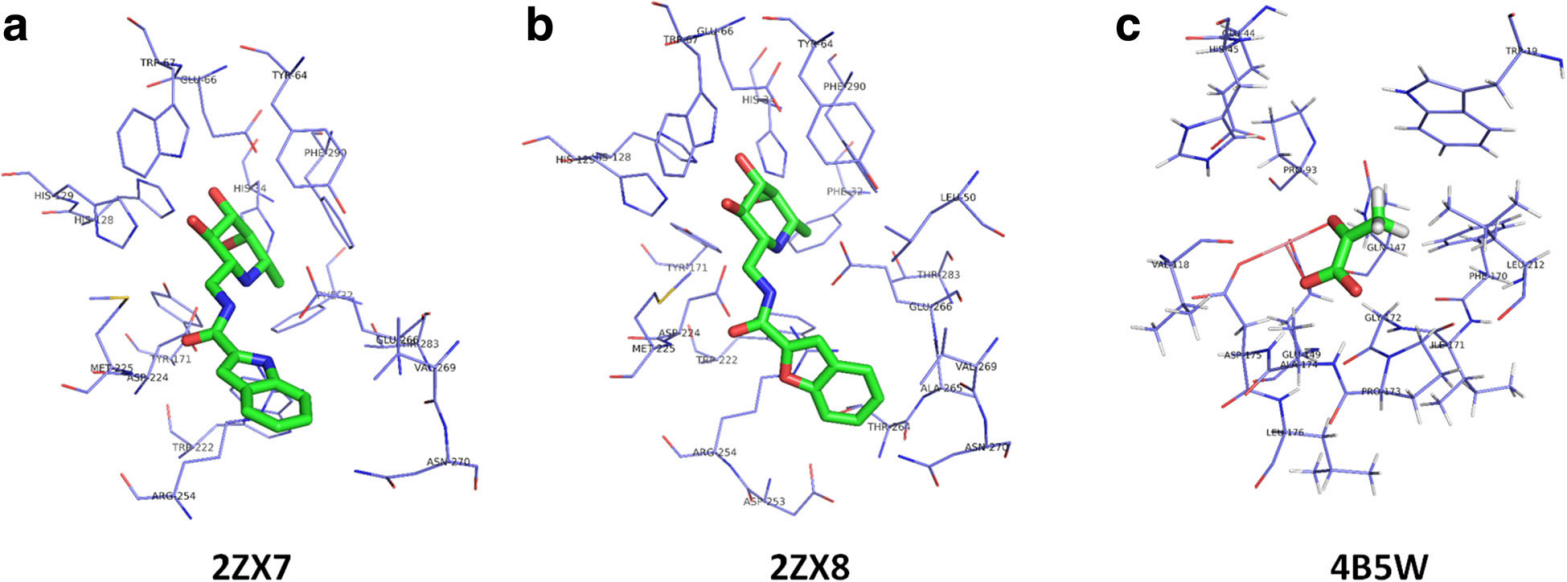
One example illustrating the different reference complexes selected by KGS2 and KGS. **a** Binding pocket on the query complex, a complex formed by α-L-fucosidase and an small-molecule inhibitor (PDB entry 2ZX7); (**b**) Binding pocket on the reference complex selected by KGS2, which is also a complex formed by α-L-fucosidase (PDB entry 2ZX8); (**c**) Binding pocket on the reference complex selected by KGS, which is a complex formed by 4-hydroxy-2-oxo-heptane-1,7-dioate aldolase (PDB entry 4B5W)

Here, we provide one example to illustrate the advantage of KGS2 over KGS in selecting a more appropriate reference complex. PDB entry 2ZX7, a complex formed by α-L-fucosidase and a small-molecule inhibitor, was chosen as the query complex (Fig. 6a). The inhibition constant (*K*_i_) of this inhibitor was reported to be 32.2 pM (−log*K*_i_ = 10.49) [64]. The binding score given by X-Score for this complex was 6.34 in log*K*_a_ units, which deviated from the true value significantly. The reference complex selected by KGS2 was PDB entry 2ZX8 (*K*_i_ = 231.4 pM; −log*K*_i_ = 9.64) [64]. This complex is also a complex formed by α-L-fucosidase, and the ligand molecule in it is a close analog to that in the query complex (Fig. 6b). On the other hand, the reference complex selected by KGS was PDB entry 4B5W (*K*_i_ = 0.47 mM; −log*K*_i_ = 3.33) [65]. This complex is formed by a different protein, i.e. 4-hydroxy-2-oxo -heptane-1,7-dioate aldolase, and the ligand molecule therein basically has nothing in common with the one in the query complex (Fig. 6c). Apparently, the reference complex selected by KGS2 resembled the query complex better. The adjusted binding score given by X-Score + KGS2 was 9.24; whereas the score given by X-Score + KGS was 4.98. In this case, a significant improvement was achieved by KGS2, where the absolute error was reduced from 4.15 to 1.25 log*K*_a_ units. In contrast, the binding score was adjusted to the wrong direction by KGS, where the absolute error was increased from 4.15 to 5.51 log*K*_a_ units.

### Performance of the standard model of KGS2 in the in situ scoring test

Besides X-Score, the other three selected scoring functions (ChemPLP, ASP, and GoldScore) were also applied to compute the entire PDBbind refined set. The statistical results between the experimental binding data and the binding scores computed by all four scoring functions are summarized in Table 2. The purpose here was to obtain the parameter *k* needed in Eq. 4 for each scoring function. The statistical results produced by those four scoring functions on the five test sets (i.e. complexes of HIV-1 protease, CA-2, BACE-1, trypsin, and CHK-1) are also summarized in Table 2. One can see that all four scoring functions demonstrated case-dependent performance, ranging from the very poor performance (*R* ≈ 0) on HIV-1 protease complexes to the more acceptable performance (*R* = 0.50 ~ 0.70) on BACE-1, trypsin, and CHK-1 complexes.

**Table 2:** Statistical results of the four selected scoring functions in the in situ scoring test

Data sets	X-Score			ChemPLP			ASP			GoldScore		
	*R* ^*a*^	*SD* ^*b*^	*k* ^*c*^	*R*	*SD*	*k*	*R*	*SD*	*k*	*R*	*SD*	*k*
The PDBbind refined set (*N* = 3446)	0.55	1.68	0.94	0.39	1.85	0.025	0.35	1.88	0.043	0.19	1.97	0.008
The PDBbind refined set (*N* = 2859)^*d*^	0.52	1.65	0.97	0.41	1.76	0.027	0.34	1.81	0.040	0.17	1.90	0.007
HIV-1 protease (*N* = 303)	0.08	1.71	---	0.01	1.73	---	−0.09	1.77	---	−0.01	1.67	---
Carbonic anhydrase 2 (*N* = 230)	0.35	1.45	---	0.40	1.43	---	0.45	1.39	---	0.37	1.45	---
Beta-secretase 1 (*N* = 223)	0.73	1.07	---	0.65	1.18	---	0.70	1.15	---	0.58	1.29	---
Beta-Trypsin (*N* = 196)	0.75	1.34	---	0.55	1.52	---	0.61	1.50	---	0.43	1.63	---
Checkpoint kinase 1 (*N* = 61)	0.63	1.23	---	0.75	1.25	---	0.81	1.28	---	0.55	1.43	---

^a^Pearson correlation coefficient between the experimental binding constants and the binding scores produced by a scoring function

^b^Standard deviation in regression (in log*K*_a_ units)

^c^Slope of the regression line, which a parameter required in Eq. 4 for KGS2 to compute adjusted binding scores

^d^This set of results were particularly derived for Variation Model 1 (see the descriptions in Preparation of the reference library)

**Fig. 7:**
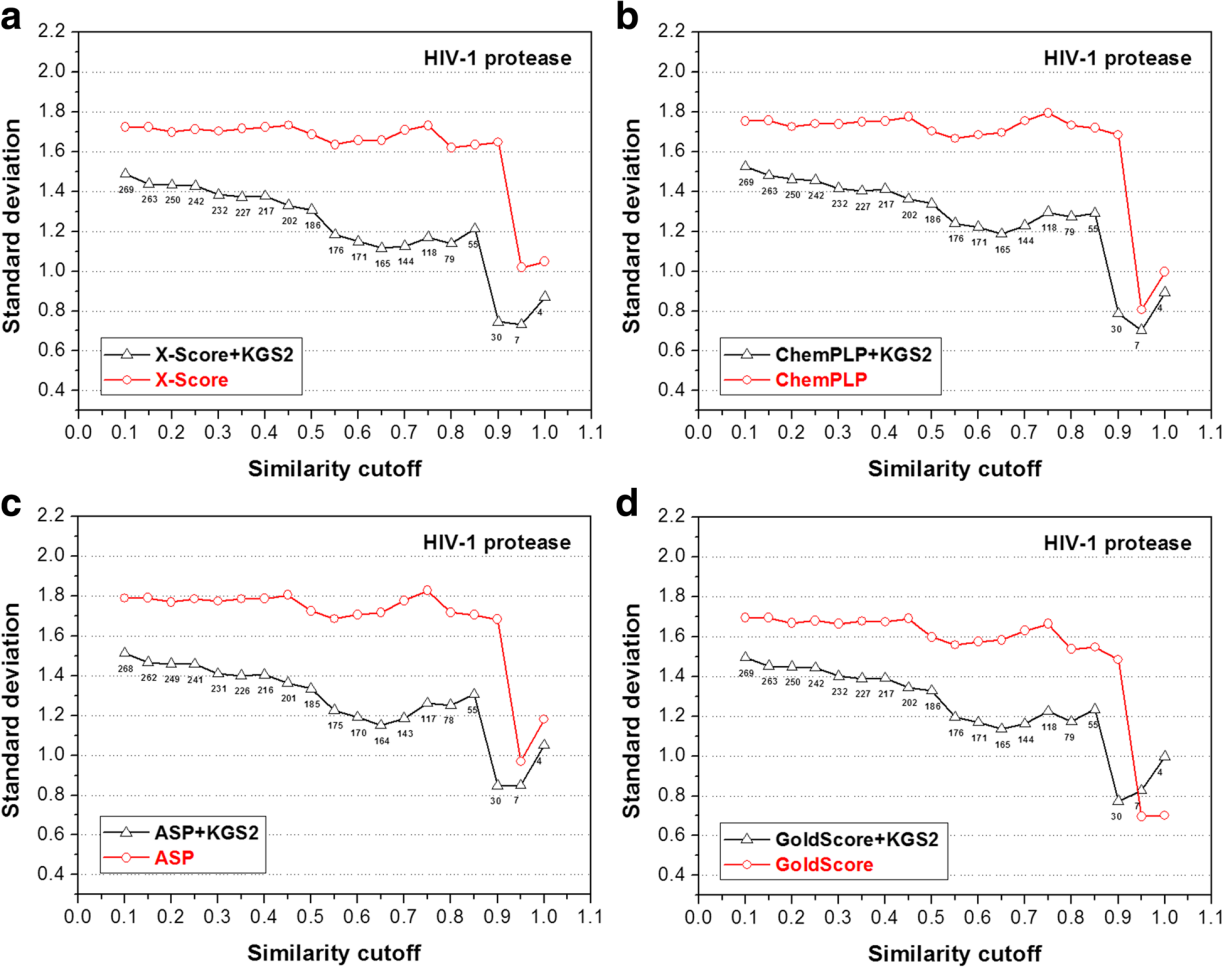
Results produced by four scoring functions, including (**a**) X-Score; **b** ChemPLP; **c** ASP; and (**d**) GoldScore, in combination with KGS2 on the HIV-1 protease test set. All annotations in this figure are similar to those used in Fig. 5. Results produced by these scoring functions in combination with KGS are given in the Additional file 1: Figure S2

**Fig. 8:**
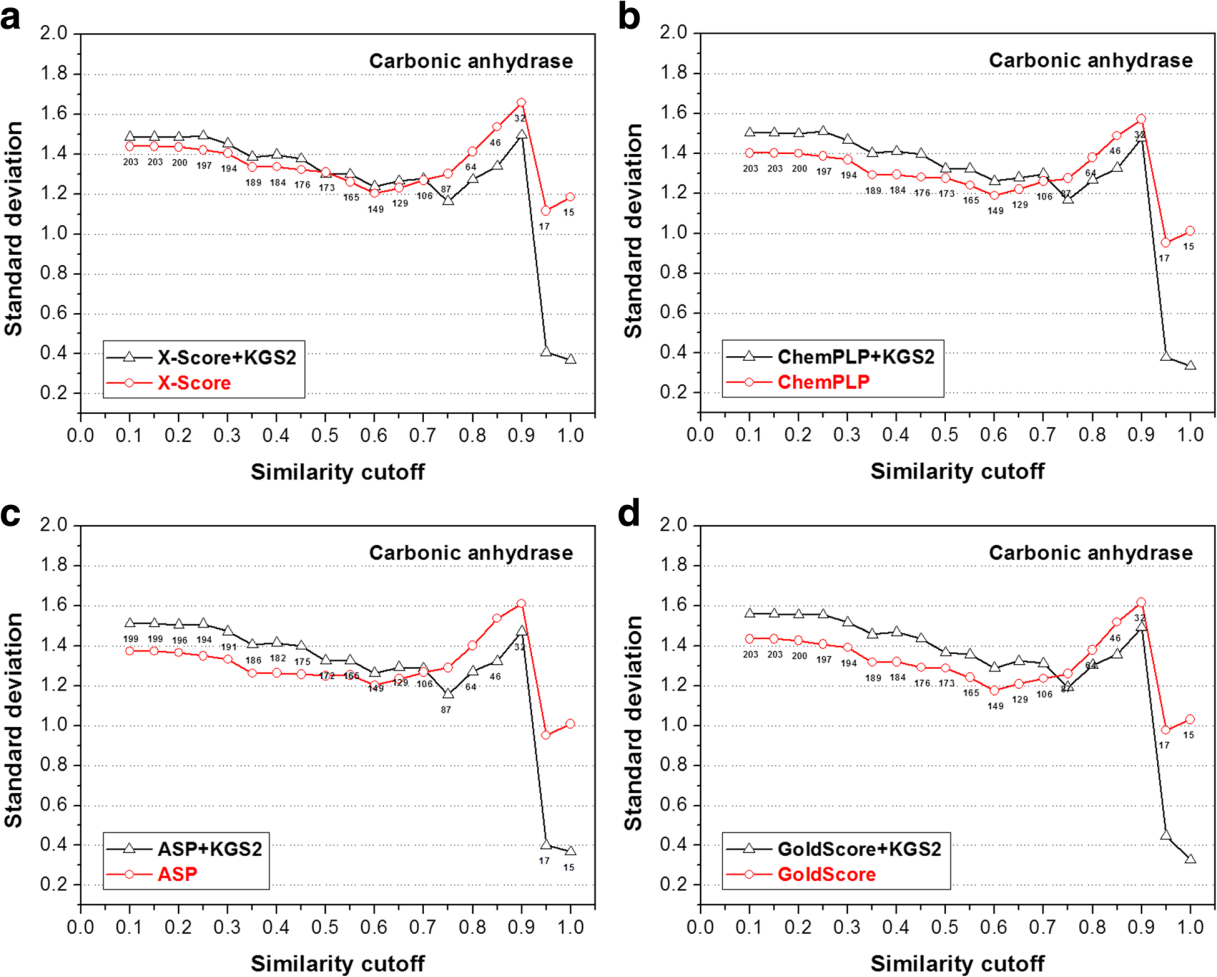
Performance of four scoring functions, including (**a**) X-Score; **b** ChemPLP; **c** ASP; and (**d**) GoldScore, in combination with KGS2 on the carbonic anhydrase 2 test set. All annotations in this figure are similar to those used in Fig. 5. Results produced by these scoring functions in combination with KGS are given in the Additional file 1: Figure S3

**Fig. 9:**
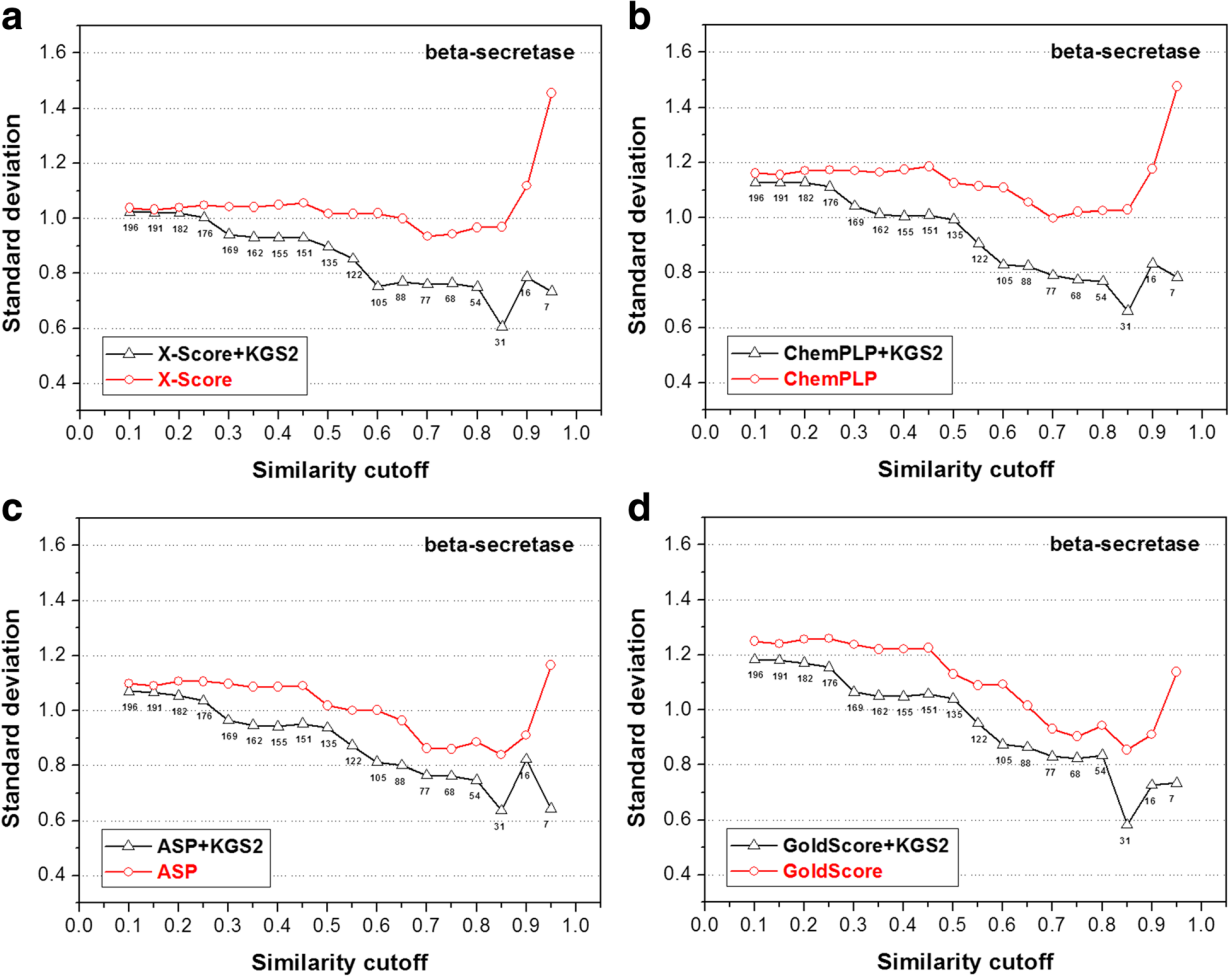
Performance of four scoring functions, including (**a**) X-Score; **b** ChemPLP; **c** ASP; and (**d**) GoldScore, in combination with KGS2 on the beta-secretase 1 test set. All annotations in this figure are similar to those used in Fig. 5. Results produced by these four scoring functions in combination with KGS are given in the Additional file 1: Figure S4

**Fig. 10:**
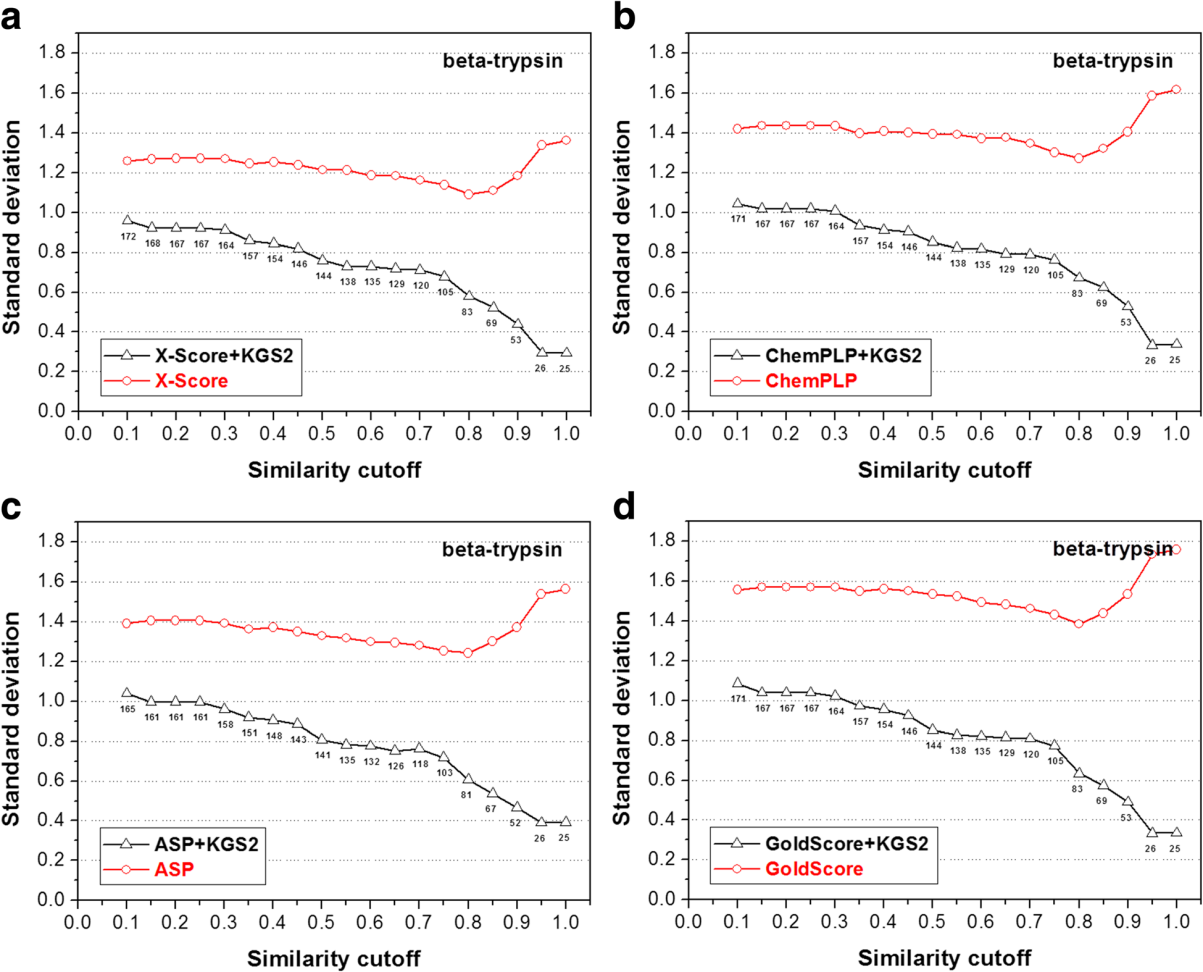
Performance of four scoring functions, including (**a**) X-Score; **b** ChemPLP; **c** ASP; and (**d**) GoldScore, in combination with KGS2 on the beta-trypsin test set. All annotations in this figure are similar to those used in Fig. 5. Results produced by these scoring functions in combination with KGS are given in the Additional file 1: Figure S5

**Fig. 11:**
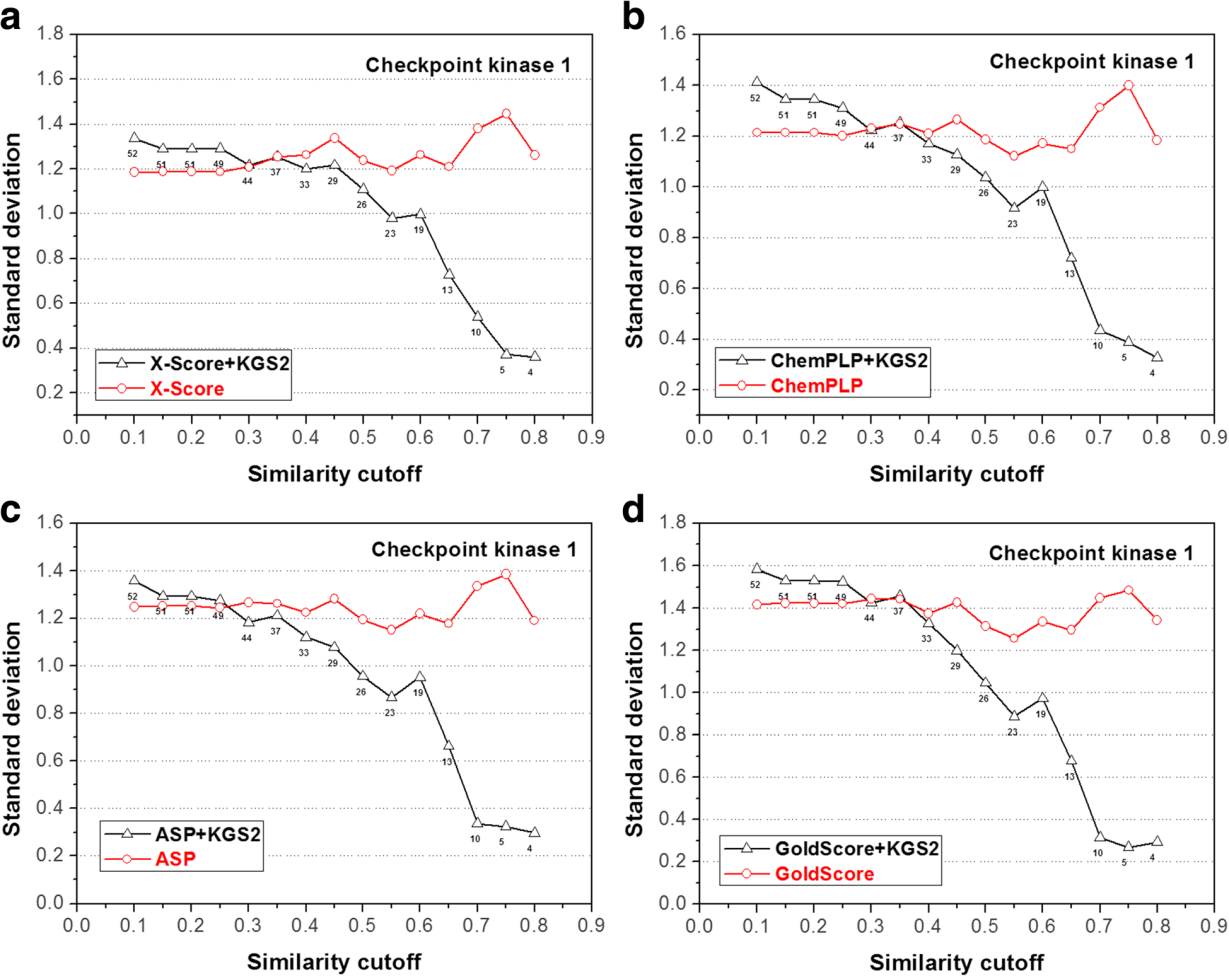
Performance of four scoring functions, including (**a**) X-Score; **b** ChemPLP; **c** ASP; and (**d**) GoldScore, in combination with KGS2 on the checkpoint kinase 1 test set. All annotations in this figure are similar to those used in Fig. 5. Results produced by these scoring functions in combination with KGS are given in the Additional file 1: Figure S6

The statistical results produced by KGS2 in combination with all four scoring functions on the HIV-1 protease test set are shown in Fig. 7. First of all, one can see that application of KGS2 resulted in more accurate binding scores for all four scoring functions. Average errors were reduced by 0.2 ~ 0.3 log*K*_a_ units even at the lowest similarity required in reference selection (i.e. *SI* ≥ 0.10). The improvement achieved by KGS2 is even more obvious at higher levels of required similarity, reaching up to 0.5 ~ 0.6 log*K*_a_ units. It should be noted that in Fig. 7 (as well as Figs. 8, 9, 10 and 11), the several data points at the far right end should be ignored because the sample size in those cases is too small for deriving any statistically meaningful conclusion. We also tested the original KGS in combination with the four scoring functions on this test set. The results are given in Additional file 1: Figure S2. An observation common to all four scoring functions is that KGS started to produce more accurate binding scores only when the similarity cutoff required in reference selection was above 0.40. In contrast, there was no such critical point for KGS2 on this test set. If the adjusted binding scores are required to be at least 0.3 log*K*_a_ units (corresponding to one-fold difference in binding constant) more accurate than those produced by standard scoring functions, KGS made it for only 29 complexes; while KGS2 made it for 232 complexes, accounting for 77% of the entire test set.

The statistical results produced by the four scoring functions in combination with KGS2 on the other four test sets (CA-2, BACE-1, beta-trypsin, and CHK-1) are shown in Figs. 8, 9, 10 and 11, respectively. Similar to the case of HIV-1 protease, the performance of KGS2 on BACE-1 (Fig. 9) and beta-trypsin (Fig. 10) was also very promising. Basically, KGS2 produced more accurate binding scores in combination with all four scoring functions regardless the similarity cutoff required in reference selection. The improvement became more obvious with higher similarity cutoffs required in reference selection. On these two test sets, application of KGS2 reduced the average error in computed binding scores below 1.0 log*K*_a_ units for a large fraction of the test set (60% ~ 100% depending on the partner scoring function).

On the contrary, KGS2 was not successful on the CA-2 test set. Application of KGS2 in this case produced essentially comparable results as standard scoring functions, i.e. no obvious improvements were observed (Fig. 8). On the CHK-1 test set, KGS2 was modestly successful (Fig. 11). One can see that there was a “critical point” for KGS2 to be effective, i.e. the similarity cutoff required in reference selection needed to be above 0.30 ~ 0.40. Above this cutoff, KGS2 produced more accurate binding scores for over half of this test set no matter which scoring function it worked with. According to Eq. 7, assuming that the size of interaction fingerprints *P* and *Q* is equal and half of their elements are in common, the similarity index between them is 0.33. This gives one a rough idea of how closely the reference complex should resemble the query complex (i.e. half of the protein-ligand interaction patterns in two complexes are in common) when KGS2 became effective in this case.

The statistical results produced by the original KGS method in combination with the four scoring functions on the CA-2, BACE-1, beta-trypsin, and CHK-1 test sets are given in Additional file 1: Figure S3-S6). Basically, KGS2 produced more accurate binding scores for a significantly larger number of samples in each test set than KGS, including the CA-2 and CHK-1 test sets where KGS2 was not very successful. In conclusion, the results obtained in our in situ scoring test again demonstrate the superior performance of KGS2 over KGS.

### Performance of three variation models in the in situ scoring test

Besides the standard model of KGS2, three variation models (see Variations of the standard model) were also examined in the in situ scoring test on the same five test sets. The purpose was to validate the algorithms implemented in KGS2 by a head-to-head comparison. Note that the similarity cutoff required in reference selection may vary between 0.1 and 1.0 when applying KGS2 as well as the three variations. For the sake of convenience, the statistical results produced by each model at the similarity cutoff of 0.35 are summarized in Table 3.

**Table 3:** Statistical results produced by the standard model of KGS2 and three variations in the in situ scoring test^*a*^

Test set	Partner scoring function	Standard model of KGS2		Variation model 1		Variation model 2		Variation model 3	
		Average reduced error^*b*^	Applicable samples	Average reduced error^*b*^	Applicable samples	Average reduced error^*b*^	Applicable samples	Average reduced error^*b*^	Applicable samples
HIV-1 protease (*N* = 303)	X-Score	0.41	227	0.41	227	−0.11	137	0.17	266
	ChemPLP	0.45	227	0.46	227	−0.12	137	0.11	266
	ASP	0.38	227	0.36	227	−0.22	137	0.08	266
	GoldScore	0.26	227	0.25	227	−0.29	137	0.03	266
Carbonic anhydrase 2 (*N* = 230)	X-Score	−0.05	189	−0.05	189	0.25	75	−0.04	203
	ChemPLP	−0.13	189	−0.13	189	0.25	75	0.00	203
	ASP	−0.10	189	−0.10	189	0.21	75	−0.02	203
	GoldScore	−0.10	189	−0.09	189	0.22	75	0.04	203
Beta-secretase 1 (*N* = 223)	X-Score	0.12	162	0.12	162	−0.09	9	−0.04	184
	ChemPLP	0.13	162	0.13	162	−0.15	9	−0.07	184
	ASP	0.14	162	0.14	162	−0.04	9	−0.07	184
	GoldScore	0.29	162	0.30	162	0.45	9	0.03	184
Beta-Trypsin (*N* = 196)	X-Score	0.33	157	0.33	157	0.37	66	0.05	164
	ChemPLP	0.47	157	0.46	157	0.55	66	0.24	164
	ASP	0.43	157	0.44	157	0.57	66	0.26	164
	GoldScore	0.59	157	0.60	157	0.71	66	0.39	164
Checkpoint kinase 1 (*N* = 61)	X-Score	0.00	37	0.00	37	−0.67	3	−0.10	48
	ChemPLP	0.00	37	0.00	37	−0.71	3	−0.00	48
	ASP	0.05	37	0.04	37	−0.70	3	0.06	48
	GoldScore	−0.01	37	−0.01	37	−0.61	3	0.07	48

^a^The similarity cutoff used in reference selection was 0.35 for all models listed in this table

^b^Average reduced error (in log*K*_a_ units) after application of KGS2, e.g. error by X-Score minus error by X-Score + KGS2. This value was computed among all applicable samples at the given similarity cutoff

As for Variation Model 1, the parameter *k* in Eq. 4 for each scoring function was derived on the PDBbind refined set after excluding the complexes that overlapped with the five test sets (i.e. 3446–587 = 2859 complexes). The statistical results of all four scoring functions produced thereby are given in the second line in Table 2. One can see that in all four cases, the regression results (including *R*_*p*_, *SD*, and *k*) obtained on the tailored refined set were very close to those obtained on the full refined set. This is understandable since only a minor fraction of the refined set (17%) was excluded here. Besides, because the refined set is a large, non-discriminatory mixture of diverse protein-ligand complexes, removing the complexes formed by any particular target protein will not cause a significant change to the genotypes/chemotypes included in this data set. The statistical results produced by Variation Model 1 on the five test sets are given in Table 3, which are almost identical in every aspect as those produced by the standard model of KGS2. Given the fact that the *k* parameters are really close in both models, this observation is not surprising. Therefore, we conclude that if KGS2 is to be evaluated on other target proteins, it is not necessary either to remove the relevant complexes from its default reference library when deriving the parameter *k* in Eq. 4.

Variation Model 2 was designed to investigate if the 3D interaction fingerprints used in KGS2 was really helpful. It is generally assumed that small-molecule compounds sharing similar 2D chemical structures tend to have similar properties or activities. Within the framework of KGS2, it is also possible to select the reference complex by simply comparing the 2D chemical structures of ligand molecules. The statistical results produced by Variation Model 2 at the similarity cutoff of 0.35 are given in Table 3; while the full results produced by Variation Model 2 can be found in the Additional file 1: Figure S7-S11. One can see that Variation Model 2 was most successful in the case of trypsin and CA-2, where it reduced error consistently by 0.3 ~ 0.7 log*K*_a_ units among applicable samples. Interestingly, Variation Model 2 was effective in the case of CA-2; whereas KGS2 was not. Nevertheless, in the case of BACE-1, the performance of Variation Model 2 varied upon the similarity cutoff as well as the partner scoring function, where no obvious trend was observed. In the case of HIV-1 protease and CHK-1, Variation Model 2 failed badly, where it actually produced worse binding scores than standard scoring functions. It is fair to conclude that the performance of the standard model of KGS2 is more robust than Variation Model 2 on those test sets. Thus, it proves that including 3D structural information in the interaction fingerprints employed by KGS2 is indeed helpful.

Variation Model 3 was designed to investigate if including the structural information from the ligand side in the interaction fingerprints employed by KGS2 was really helpful. This is a valid question because some forms of 3D interaction fingerprints are designed based on protein structures only [45]. The results produced by Variation Model 3 at the similarity cutoff of 0.35 are given in Table 3; while the full results produced by Variation Model 3 can be found in the Additional file 1: Figure S12-S16. Generally speaking, Variation Model 3 was effective in the case of HIV-1 protease and trypsin, where it yielded reduced errors of 0.1 ~ 0.4 log*K*_a_ units depending on the applied similarity cutoff as well as the partner scoring function. However, it was not effective on the other three test sets. Unlike Variation Model 2, it did not produce worse binding scores in those cases. Compared to the standard model, the overall performance of Variation Model 3 is consistently inferior on each test set. Thus, we conclude that the 3D interaction fingerprints employed by KGS2 is more effective than the algorithm that considers protein structures only.

### Performance in the molecular docking tests

In our in situ scoring test, the binding affinity of a protein-ligand complex was computed based on its experimentally resolved 3D structure. This is of course not practical in reality because obtaining a complex structure is far more difficult than conducting a decent binding assay. Thus, the in situ scoring test in our study actually serves as a proof-of-concept. Our second type of test was designed to mimic the reality in structure-based drug design more closely. Assuming that the binding affinity data of a set of ligand molecules have been measured, now a molecular modeler chooses to employ molecular docking to derive the binding modes of these compounds and also attempt to compute their binding affinities. Here, the purpose is to interpret the structure-activity relationship of these compounds to provide a guidance for further structural optimization. The quality of such a workflow is typically judged by how well the computed binding data explain experimental observations.

The statistical results produced by four scoring functions on the four selected test sets are summarized in Table 4. First of all, one can see that the standard docking/scoring methods were not very successful in reproducing the structure-activity relationships on these four test sets. According to the classical theory of statistics [66], a correlation coefficient *R* > 0.45 (i.e. *R* ^2^ > 0.20) indicates a statistically significant correlation for a data set of 12 ~ 15 samples at the 90% confidence level. By this standard, only one out of the total 16 trials (four scoring functions × four test sets = 16 individual trials) achieved a significant correlation, i.e. ChemPLP on the HIV-1 protease test set (*R* ^2^ = 0.244). This result demonstrates again that binding affinity prediction is still a very challenging task for today’s standard docking/scoring methods.

**Table 4:** Performance of four scoring functions in combination with KGS2 in the molecular docking test

Scoring scheme	Squared correlation coefficient (*R* ^2^) between experimental and computed binding data			
	HIV-1 protease (*N* = 12)	Carbonic anhydrase 2 (*N* = 15)	Beta-secretase 1 (*N* = 14)	Checkpoint kinase 1 (*N* = 15)
X-Score	0.000	0.194	0.055	0.005
X-Score + KGS2	0.012	0.195	0.028	0.149
ChemPLP	0.244	0.004	0.058	0.077
ChemPLP + KGS2	0.364	0.041	0.000	0.239
ASP	0.162	0.054	0.016	0.120
ASP + KGS2	0.168	0.123	0.000	0.246
GoldScore	0.086	0.086	0.019	0.023
GoldScore + KGS2	0.238	0.092	0.082	0.216

As shown in Table 4, the performance of KGS2 here was dependent on the target protein as well as the partner scoring function. In the case of HIV-1 protease, KGS2 led to improved results with ChemPLP and GoldScore but not the other two scoring functions. In the case of CA-2 and BACE-1, KGS2 had no obvious effect with all four scoring functions. Relatively successful results were obtained in the case of CHK-1, where KGS2 led to improved results with all four scoring functions. If still taking *R* > 0.45 as the threshold, the total number of trials where a significant correlation was achieved increased from one to five after application of KGS2, including ChemPLP and GoldScore on HIV-1 protease, ChemPLP, ASP, and GoldScore on CHK-1.

**Fig. 12:**
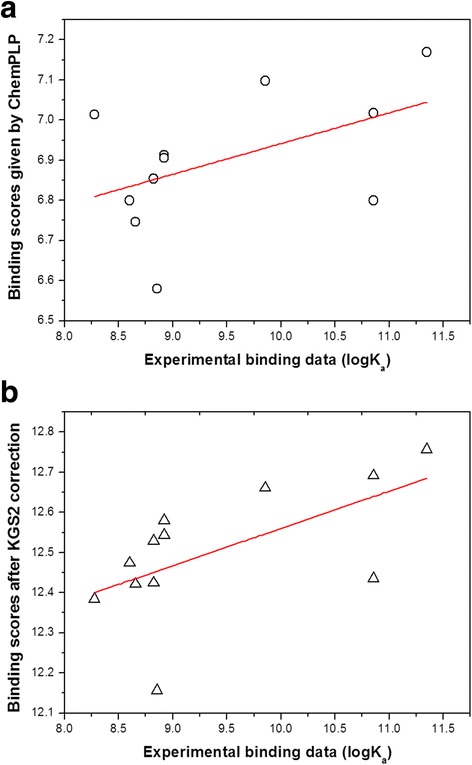
Correlation between the experimental and computed binding data on the HIV-1 protease data set in the molecular docking test. **a** Given by ChemPLP alone: *R* ^2^ = 0.244; **b** Given by ChemPLP + KGS2: *R* ^2^ = 0.364

**Fig. 13:**
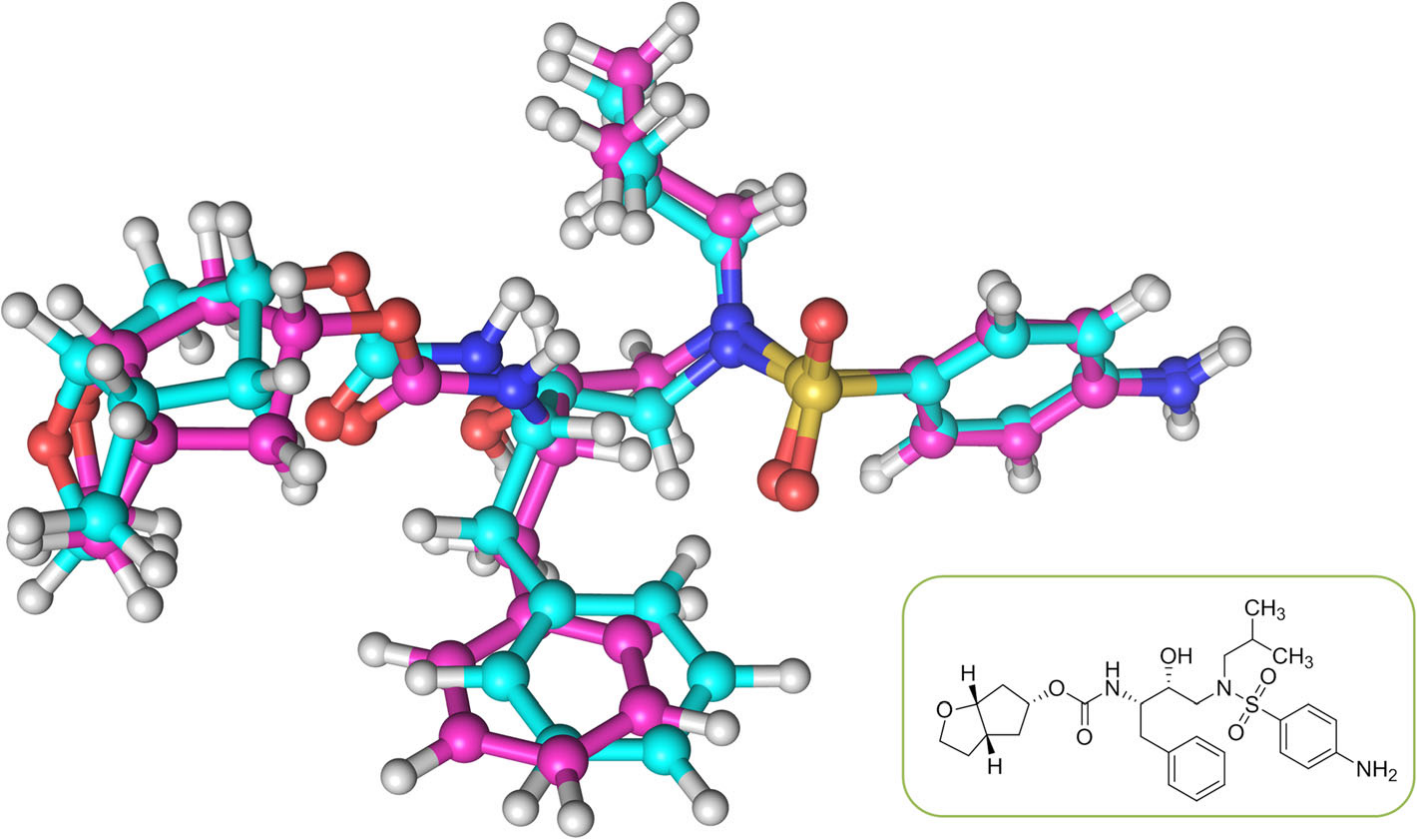
Superposition of the binding pose selected by ChemPLP alone (in *cyan*) and the binding pose selected by ChemPLP + KGS2 (in *magenta*) for ligand BDBM13924 in the HIV-1 protease test set. The target protein in the complex is concealed in this figure

Here, we further discuss the results produced by ChemPLP + KGS2 on the test set of HIV-1 protease as an example. Basic information of this data set as well as the computed results given by ChemPLP and ChemPLP + KGS2 are given in Additional file 1: Table S2. In this case, ChemPLP alone produced *R* ^2^ = 0.244 (Fig. 12a). After application of KGS2, all samples in this data set except two outliers were lined up quite well, where the correlation was improved to *R* ^2^ = 0.364 (Fig. 12b). The reason is that a different top-ranked binding pose for each ligand molecule could be selected by KGS2. One example is shown in Fig. 13, which illustrates the top-ranked binding pose selected by ChemPLP alone and the one by ChemPLP + KGS2. One can see that these two binding poses differ from each other in the benzyl moiety and the bicyclic hexahydro-2H-cyclopenta furan moiety; while the rest parts of this ligand molecule basically overlap. The overall RMSD value between these two binding poses is only 0.42 Å. It is interesting to observe that the 3D interaction fingerprints employed by KGS2 are able to distinguish such subtle conformational difference. This observation demonstrates why KGS2 may be used to re-rank the binding poses generated by a conventional molecular docking method.

Generally speaking, KGS2 demonstrated only modest success in this set of molecular docking tests. But this can be attributed to an obvious reason: As indicated by the very low *R* values, the standard docking/scoring methods employed by us were not very successful in deriving the structure-activity relationships of those four test sets at the first place. KGS2 is essentially a method for making corrections to the outcomes of a standard scoring function. If the scoring function itself produces terrible results, KGS2 cannot magically solve the problem because the starting point given to it is too poor. In our opinion, this is the primary limitation for applying KGS2 in practice.

It needs to be mentioned, however, that the disappointing performance of GOLD on those four test sets does not imply that it will always fail in such a scenario. In fact, numerous successful applications of GOLD have been reported in literature. If the ligand binding poses were prepared more carefully by fine-tuning the adjustable parameters used in GOLD or even employing molecular dynamics simulations, binding affinities of those several sets of ligand molecules may be reproduced better. In short, the molecular docking results in this set of test were obtained in a straightforward, “pain free” manner, which intended to mimic the routine jobs done by a molecular modeler. Yet, application of KGS2 still led to some encouraging improvements. We suggest that one should apply KGS2 in combination with a capable computational method that can generate reliable ligand binding modes, and in turn KGS2 will be more likely to produce more accurate predictions of ligand binding affinity.

### Advantages and limitations of the KGS2 method

In this study, four selected scoring functions were evaluated in in situ scoring and molecular docking tests. Generally speaking, those scoring functions did not reproduce known ligand binding affinities well in both sets of tests (Tables 2 and 4). Besides, our results indicate that their performance is largely case-dependent. Customized scoring functions offer an appealing approach to overcome this difficulty. However, development and application of customized scoring functions are confronted with certain technical inconvenience. Thus, alternative approaches are also welcome by the end users in this field.

Our KGS2 method is developed as a convenient “add-on” to make current all-purpose scoring functions work as customized scoring functions. An obvious technical advantage of KGS2 is that in theory, it may be applied in combination with any scoring function (or other types of scoring methods). It is demonstrated in our study that KGS2 indeed worked with different types of scoring functions in a non-discriminatory manner to produce more accurate binding scores. Besides, one does not need to re-engineer a scoring function before applying KGS2. The only important input required from the user side is a “reference library”, i.e. a data set of protein-ligand complexes with known 3D structures and experimental binding data. Ideally, this reference library should consist of a sufficient number of protein-ligand complexes formed by the same target protein as the query. Such a library may be compiled from public data resources, such as the PDBbind database. Users in pharmaceutical companies have the privilege to utilize their internal data for this purpose. Use of an external reference library is more flexible when one needs to deal with different target proteins: Once a different target protein is under consideration, one just needs to replace the reference library. If one does not bother to switch between different reference libraries, a comprehensive reference library containing diverse protein-ligand complexes may be employed as default.

Another advantage of KGS2 lies in the fact that KGS2 is essentially an interpolation method. More accurate predictions can be made through interpolation if more data points are known in the problem space. Knowledge of protein-ligand complex structures and binding data is being accumulated rapidly. For example, the total number of protein-ligand complexes recorded in the PDBbind database increases by 10% each year. Consequently, larger and finer references libraries may be complied for KGS2, which in turn is expected to produce more accurate results based on them. Customized scoring functions of course can utilize the increasing knowledge of protein-ligand complex structures and binding data as well, for example, by conducting parameterization on larger data sets. However, the accuracy of customized scoring functions will not improve in a proportional manner with the expansion of data sets. In contrast, an interpolation method benefits from data expansion more directly.

The limitations of KGS2 also need to be mentioned clearly. First, KGS2 will not be useful if an appropriate reference complex cannot be found for the query complex. It is usually not a problem for a well-established target protein where abundant complex structures and binding data have been accumulated. However, if one deals with a rare or new target protein, it is possible that KGS2 is not applicable at all. The second limitation is that our definition of interaction units does not account for some special components, such as structural water molecules and metal ions located in the binding pocket. Those components are important in some cases because they bridge the interactions between protein and ligand. Ignoring such components certainly has a negative impact. For example, KGS2 was not very successful in the case of CA-2 in our in situ scoring test, where CA-2 is the only one among the five selected target proteins that has a metal ion inside its binding pocket. In addition, by our current algorithm each interaction pattern makes an equal contribution when two complex structures are compared (Equation 7). But this is not always true. For example, a hydrogen bond is presumably more critical than two contacting hydrophobic groups. Within the theoretical framework of KGS2, there is still plenty of room for improvements.

Finally, the potential applications of KGS2 in structure-based drug design should be discussed. As indicated by its name, i.e. “Knowledge-Guided Scoring”, KGS2 is designed primarily as a “scoring” method for binding affinity prediction. As demonstrated in the second set of tests conducted in this study (i.e. the molecular docking test), KGS2 can be used in combination with standard docking/scoring protocols to interpret the structure-activity relationship of a congeneric set of lead compounds. The outcome of such effort provides a guidance for further structural optimization of those compounds, which is particularly useful when experimental techniques cannot resolve the desired protein-ligand complex structures in a timely manner. More rigorous methods, such as the free energy perturbation (FEP) method [67, 68] and the MM-GB/SA (or MM-PB/SA) method [69, 70], are often employed for the same type of task as well. But those sampling-based methods are computationally more expensive. In contrast, our KGS2 method is designed to work with standard scoring functions to provide the users quick feedbacks. Currently, we do not recommend the users to apply KGS2 to virtual screening jobs since it has not been tuned toward this type of task yet.

## Conclusions

Our KGS2 method can serve as a convenient “add-on” to enhance the performance of current scoring functions in binding affinity prediction without the need to re-engineer them. As compared to the original KGS method, the major improvement in KGS2 is the introduction of 3D protein-ligand interaction fingerprints as the basis of comparing complex structures. In this study, KGS2 was first validated on a set of in situ scoring tests, where a non-discriminatory set of protein-ligand complexes and five sets of complexes formed by HIV-1 protease, CA-2, BACE-1, beta-trypsin, and CHK-1, respectively. The results obtained in this test indicated that KGS2 consistently outperformed KGS in all cases. The performance of KGS2 was also more robust than two variations (i.e. Variation Model 2 and Variation Model 3) which employed different algorithms in reference selection. Moreover, KGS2 produced more accurate binding scores in combination with all four different scoring functions (ChemPLP, ASP, GoldScore, and X-Score) on four out of five test sets. These results verify our assumption that KGS2 in principle can work with any scoring function and is applicable to various target proteins.

In this study, a more challenging set of molecular docking tests were also conducted on four sets of ligands of HIV-1 protease, CA-2, BACE-1, and CHK-1, respectively. The four scoring functions, if applied alone, achieved rather limited success on those data sets. Here, application of KGS2 did not lead to an improvement as obviously as in the in situ scoring test. However, it did increase the number of significant correlation from one to five out of a total of 16 trials. As the in situ scoring test serves as a proof-of-concept, this test demonstrates the possible application of KGS2 in practice, i.e. interpreting the structure-activity relationship of congeneric lead compounds to guide further structural optimization. The potential of KGS2 in other types of tasks, such as virtual screening and de novo design, has yet to be explored.

## Additional file

Additional file 1: Addtional tables (**Table S1**-**S2**) and figures (**Figure S1**-**S16**) mentioned in the main text. (PDF 3600 kb)

## Abbreviations

FEP: Free energy perturbation
KGS: Knowledge-guided scoring
