# Supplementary material for: Enhance the performance of current scoring functions with the aid of 3D protein-ligand interaction fingerprints
Source: BMC Bioinformatics. 2017 Jul 18;18:343. doi: 10.1186/s12859-017-1750-5 (PMC5516336; doi:10.1186/s12859-017-1750-5)
Supplement: Additional file 1: — Addtional tables (Table S1-S2) and figures (Figure S1-S16) mentioned in the main text. (PDF 3600 kb) [file 12859_2017_1750_MOESM1_ESM.pdf]

Additional file 1

Enhance the Performance of Current Scoring Functions with the Aid of 3D Protein-Ligand Interaction Fingerprints

Jie Liu,[a] Minyi Su,[a] Zhihai Liu,[a] Jie Li,[a] Yan Li,[a,]* Renxiao Wang [a,b,]*

[a] *State Key Laboratory of Bioorganic and Natural Products Chemistry, Collaborative Innovation Center of Chemistry for Life Sciences, Shanghai Institute of Organic Chemistry, Chinese Academy of Sciences, 345 Lingling Road, Shanghai 200032, China*

[b] *State Key Laboratory of Quality Research in Chinese Medicine, Macau Institute for Applied Research in Medicine and Health, Macau University of Science and Technology, Macau, People's Republic of China*

* To whom all correspondence should be addressed: kathyli@sioc.ac.cn; wangrx@sioc.ac.cn;

TABLE OF CONTENTS

Table S1. PDB codes of the protein-ligand complexes in the five test sets

Table S2. Basic information of the HIV-1 protease data set used in the molecular docking test

Figure S1. Distributions of the experimental binding constants of the protein-ligand complexes in (a) the PDBbind refined set version 2014; (b) The HIV-1 protease test set; (c) The carbonic anhydrase 2 test set; (d) The beta-secretase test set; (e) The beta-trypsin test set. (f) The checkpoint kinase 1 test set.

Figure S2. Results produced by (a) X-Score, (b) ChemPLP, (c) ASP, and (d) GoldScore in couple with KGS on the HIV-1 protease test set. All annotations in this figure are similar to those used in Figure 5 in the main text.

Figure S3. Results produced by (a) X-Score, (b) ChemPLP, (c) ASP, and (d) GoldScore in couple with KGS on the carbonic anhydrase 2 test set. All annotations in this figure are similar to those used in Figure 5 in the main text.

Figure S4. Results produced by (a) X-Score, (b) ChemPLP, (c) ASP, and (d) GoldScore in couple with KGS on the beta-secretase 1 test set. All annotations in this figure are similar to those used in Figure 5 in the main text.

Figure S5. Results produced by (a) X-Score, (b) ChemPLP, (c) ASP, and (d) GoldScore in couple with KGS on the beta-trypsin test set. All annotations in this figure are similar to those used in Figure 5 in the main text.

Figure S6. Results produced by (a) X-Score, (b) ChemPLP, (c) ASP, and (d) GoldScore in couple with KGS on the checkpoint kinase 1 test set. All annotations in this figure are similar to those used in Figure 5 in the main text.

Figure S7. Results produced by (a) X-Score, (b) ChemPLP, (c) ASP, and (d) GoldScore in couple with Variation Model 2 on the HIV-1 protease test set. All annotations in this figure are similar to those used in Figure 5 in the main text.

Figure S8. Results produced by (a) X-Score, (b) ChemPLP, (c) ASP, and (d) GoldScore in couple with Variation Model 2 on the carbonic anhydrase 2 test set. All annotations in this figure are similar to those used in Figure 5 in the main text.

Figure S9. Results produced by (a) X-Score, (b) ChemPLP, (c) ASP, and (d) GoldScore in couple with Variation Model 2 on the beta-secretase 1 test set. All annotations in this figure are similar to those used in Figure 5 in the main text.

Figure S10. Results produced by (a) X-Score, (b) ChemPLP, (c) ASP, and (d) GoldScore in couple with Variation Model 2 on the beta-trypsin test set. All annotations in this figure are similar to those used in Figure 5 in the main text.

Figure S11. Results produced by (a) X-Score, (b) ChemPLP, (c) ASP, and (d) GoldScore in couple with Variation Model 2 on the checkpoint kinase 1 test set. All annotations in this figure are similar to those used in Figure 5 in the main text.

Figure S12. Results produced by (a) X-Score, (b) ChemPLP, (c) ASP, and (d) GoldScore in couple with Variation Model 3 on the HIV-1 protease test set. All annotations in this figure are similar to those used in Figure 5 in the main text.

Figure S13. Results produced by (a) X-Score, (b) ChemPLP, (c) ASP, and (d) GoldScore in couple with Variation Model 3 on the carbonic anhydrase 2 test set. All annotations in this figure are similar to those used in Figure 5 in the main text.

Figure S14. Results produced by (a) X-Score, (b) ChemPLP, (c) ASP, and (d) GoldScore in couple with Variation Model 3 on the beta-secretase 1 test set. All annotations in this figure are similar to those used in Figure 5 in the main text.

Figure S15. Results produced by (a) X-Score, (b) ChemPLP, (c) ASP, and (d) GoldScore in couple with Variation Model 3 on the beta-trypsin test set. All annotations in this figure are similar to those used in Figure 5 in the main text.

Figure S16. Results produced by (a) X-Score, (b) ChemPLP, (c) ASP, and (d) GoldScore in couple with Variation Model 3 on the checkpoint kinase 1 test set. All annotations in this figure are similar to those used in Figure 5 in the main text.

Table S1. PDB codes of the protein-ligand complexes in the four test sets

---

HIV-1 protease complexes (N = 304)

1A30, 1A94, 1A9M, 1AAQ, 1AID, 1AJV, 1AJX, 1B6J, 1B6K, 1B6L, 1B6M, 1BV7, 1BV9, 1BWA, 1BWB, 1C70, 1CPI, 1D4H, 1D4I, 1D4J, 1D4K, 1D4L, 1D4Y, 1DIF, 1DMP, 1EBW, 1EBY, 1EBZ, 1EC0, 1EC1, 1EC2, 1EC3, 1G2K, 1G35, 1GNM, 1GNN, 1GNO, 1HBV, 1HEF, 1HEG, 1HIH, 1HOS, 1HPO, 1HPS, 1HPV, 1HPX, 1HSG, 1HTF, 1HTG, 1HVH, 1HVI, 1HVJ, 1HVK, 1HVL, 1HVR, 1HVS, 1HWR, 1HXB, 1HXW, 1IIQ, 1IZH, 1IZI, 1KZK, 1LZQ, 1M0B, 1MES, 1MET, 1MEU, 1MRW, 1MSM, 1MTR, 1NH0, 1NPA, 1NPV, 1NPW, 1ODY, 1OHR, 1PRO, 1QBR, 1QBS, 1QBT, 1QBU, 1SBG, 1SDT, 1SDU, 1SDV, 1TCX, 1VIJ, 1VIK, 1W5V, 1W5W, 1W5X, 1W5Y, 1Z1H, 1Z1R, 1ZP8, 1ZPA, 1ZSF, 1ZSR, 2AOC, 2AOD, 2AOE, 2AOG, 2AQU, 2AVM, 2AVO, 2AVQ, 2AVS, 2AVV, 2BBB, 2BPV, 2BPY, 2BQV, 2CEJ, 2CEM, 2CEN, 2F3K, 2F80, 2F81, 2F8G, 2FGU, 2FGV, 2FLE, 2HB3, 2HS1, 2HS2, 2I0A, 2I0D, 2I4D, 2I4U, 2I4V, 2I4W, 2I4X, 2IDW, 2IEN, 2IEO, 2NMY, 2NMZ, 2NNK, 2NNP, 2O4K, 2O4L, 2O4N, 2O4P, 2O4S, 2P3B, 2PK5, 2PK6, 2PQZ, 2PSU, 2PSV, 2PWC, 2PWR, 2PYM, 2PYN, 2Q54, 2Q55, 2Q5K, 2Q63, 2Q64, 2QCI, 2QD6, 2QD7, 2QD8, 2QHY, 2QHZ, 2QI0, 2QI1, 2QI3, 2QI4, 2QI5, 2QI6, 2QI7, 2QNN, 2QNP, 2QNQ, 2R38, 2R3T, 2R3W, 2R43, 2UXZ, 2UY0, 2WKZ, 2WL0, 2XYE, 2XYF, 2Z4O, 3A2O, 3AID, 3BGB, 3BGC, 3BVA, 3BVB, 3BXS, 3CKT, 3CYW, 3CYX, 3D1X, 3D1Y, 3D1Z, 3D20, 3DJK, 3DK1, 3EKV, 3EKX, 3EKY, 3EL1, 3EL4, 3EL9, 3GI4, 3GI5, 3GI6, 3H5B, 3I6O, 3I7E, 3KDB, 3KDC, 3KDD, 3KFN, 3LZV, 3M9F, 3MXD, 3MXE, 3NU3, 3NU4, 3NU5, 3NU6, 3NU9, 3NUJ, 3NUO, 3OK9, 3PWM, 3QAA, 3ST5, 3TH9, 3TKW, 3TLH, 3VF5, 3VF7, 3VFA, 3VFB, 4A6B, 4A6C, 4DFG, 4DJO, 4DJP, 4DJQ, 4DJR, 4PHV, 5HVP, 7UPJ, 1BDQ, 1K6C, 1K6P, 1K6T, 1K6V, 1MRX, 1MSN, 1SGU, 1SH9, 1T7J, 2AOF, 2AOI, 2AZC, 2FDD, 2NXL, 2NXM, 2P3A, 2P3C, 2R5P, 2RKF, 2RKG, 3EKP, 3EKQ, 3EKT, 3EKW, 3EL0, 3EL5, 3GGU, 3LZS, 3LZU, 4LL3, 3S43, 3S53, 3S54, 3S56, 3SPK, 3T3C, 3U7S, 3ZPT, 3ZPU, 4A4Q, 4EP2, 4GYE, 4GZF, 4HDB, 4HDF, 4HDP, 4HE9, 4HEG, 4HLA, 4I8W, 4I8Z, 4KB9

Carbonic anhydrase complexes (N = 230)

1A42, 1AVN, 1AZM, 1BCD, 1BN1, 1BN3, 1BN4, 1BNM, 1BNN, 1BNQ, 1BNT, 1BNU, 1BNV, 1BNW, 1CAM, 1CIL, 1CIM, 1CIN, 1CNW, 1CNX, 1CNY, 1EOU, 1G1D, 1G45, 1G46, 1G48, 1G4J, 1G4O, 1G52, 1G53, 1G54, 1H4N, 1I8Z, 1I90, 1I91, 1I9L, 1I9M, 1I9N, 1I9O, 1I9P, 1I9Q, 1IF7, 1IF8, 1JD0, 1KWQ, 1KWR, 1OKL, 1OQ5, 1TTM, 1XPZ, 1XQ0, 1YDA, 1YDB, 1YDD, 1Z9Y, 1ZE8, 1ZFK, 1ZFQ, 1ZGE, 1ZSB, 2AW1, 2GD8, 2EZ7, 2F14, 2FOU, 2FOV, 2FOY, 2H15, 2H4N, 2HD6, 2HL4, 2HNC, 2HOC, 2IT4, 2NMX, 2NN1, 2NN7, 2NNG, 2NNO, 2NNS, 2NNV, 2O4Z, 2POU, 2POV, 2POW, 2Q1Q, 2Q38, 2QO8, 2QOA, 2WD3, 2WEG, 2WEH, 2WEJ, 2WEO, 2X7S, 2X7T, 2X7U, 3C7P, 3CYU, 3CZV, 3D8W, 3D9Z, 3DAZ, 3DD0, 3DD8, 3MDZ, 3EFT, 3F7B, 3F7U, 3F8E, 3FFP, 3FW3, 3HKN, 3HKQ, 3HKT, 3HKU, 3HS4, 3IBI, 3IBL, 3IBN, 3IBU, 3IEO, 3IGP, 3K2F, 3KIG, 3KWA, 3LXE, 3M1J, 3M1K, 3M3X, 3M40, 3M5E, 3M67, 3M96, 3MHI, 3MHL, 3MHM, 3MHO, 3ML2, 3MMF, 3MNA, 3MNU, 3MYQ, 3MZC, 3N0N, 3N2P, 3N3J, 3N4B, 3NB5, 3NI5, 3OIK, 3OIL, 3OIM, 3OKU, 3OKV, 3OY0, 3OYQ, 3OYS, 3P3H, 3P3J, 3P44, 3P4V, 3P55, 3P58, 3P5L, 3PO6, 3R16, 3R17, 3RJ7, 3SAX, 3SBH, 3SBI, 3RYJ, 3RYV, 3RYX, 3RYY, 3RYZ, 3RZ0, 3RZ1, 3RZ5, 3RZ7, 3RZ8, 3S71, 3S72, 3S73, 3S74, 3S75, 3S76, 3S77, 3S78, 3S8X, 3S9T, 3SAP, 3T5U, 3T82, 3T83, 3T84, 3T85, 3UCJ, 3V5G, 3V7X, 3VBD, 3ZNC, 3ZP9, 4BCW, 4E3D, 4E3F, 4E3G, 4E3H, 4E49, 4E4A, 4G0C, 4HT0,

4HT2, 4HU1, 4IWZ, 4JSA, 4JSS, 4JSZ, 4KNI, 4KNJ, 4KNM, 4KNN, 4KP5, 4KP8, 4M2R, 4M2U, 4M2V, 4M2W, 4MO8

BACE complexes (N = 223)

1FKN, 1M4H, 1TQF, 1YM2, 1YM4, 2B8L, 2B8V, 2F3E, 2F3F, 2FDP, 2G94, 2HIZ, 2HM1, 2IQG, 2IRZ, 2IS0, 2OAH, 2OHP, 2OHQ, 2OHR, 2OHS, 2OHT, 2OHU, 2P4J, 2P83, 2P8H, 2PH6, 2PH8, 2Q11, 2Q15, 2QK5, 2QMD, 2QMF, 2QMG, 2QP8, 2QU3, 2QZK, 2QZL, 2VA6, 2VA7, 2VIE, 2VIY, 2VIZ, 2VJ6, 2VJ7, 2VJ9, 2VKM, 2VNM, 2VNN, 2WEZ, 2WF0, 2WF1, 2WF2, 2WF3, 2WF4, 2XFI, 2XFJ, 2XFK, 2ZDZ, 2ZE1, 3BRA, 3BUF, 3BUG, 3BUH, 3CIB, 3CIC, 3CID, 3CKP, 3DUY, 3DV1, 3DV5, 3EXO, 3FKT, 3HVG, 3HW1, 3I25, 3IGB, 3IN3, 3IN4, 3IND, 3INE, 3INF, 3INH, 3IVH, 3IVI, 3IXJ, 3IXK, 3K5C, 3K5F, 3K5G, 3KMX, 3KMY, 3KN0, 3L38, 3L3A, 3L58, 3L59, 3L5B, 3L5C, 3L5D, 3L5E, 3L5F, 3LHG, 3LNK, 3LPI, 3LPJ, 3LPK, 3MSJ, 3MSK, 3MSL, 3NSH, 3OHF, 3OHH, 3OOZ, 3PI5, 3QBH, 3RSV, 3RSX, 3RU1, 3S7L, 3S7M, 3TPP, 3U6A, 3UDH, 3UDJ, 3UDM, 3UDN, 3UDP, 3UDR, 3UDY, 3UFL, 3UQP, 3VEU, 3VF3, 3VG1, 3VV6, 3VV7, 3VV8, 4ACU, 4ACX, 4AZY, 4B00, 4B1C, 4B1D, 4D88, 4D89, 4DH6, 4DI2, 4DJU, 4DJV, 4DJW, 4DJX, 4DJY, 4DPF, 4DPI, 4DUS, 4EWO, 4EXG, 4FM7, 4FM8, 4FRI, 4FRJ, 4FRK, 4FRS, 4FSL, 4GID, 4H1E, 4H3F, 4H3G, 4H3I, 4H3J, 4HA5, 3QI1, 3WB4, 3WB5, 3ZMG, 3ZOV, 4B05, 4B70, 4B72, 4B77, 4B78, 4BEK, 4BFD, 4D83, 4D8C, 4DV9, 4DVF, 4FGX, 4FS4, 4HZT, 4I0D, 4I0F, 4I10, 4I11, 4I12, 4I1C, 4IVT, 4J0P, 4J0T, 4J0V, 4J0Y, 4J0Z, 4J17, 4J1C, 4J1E, 4J1F, 4J1H, 4J1I, 4J1K, 4JOO, 4JP9, 4JPC, 4JPE, 4K8S, 4K9H, 4KE0, 4KE1, 4L7G, 4LC7, 4LXA, 4LXK, 4LXM

Trypsin complexes (N = 196)

1AQ7, 1AUJ, 1BJU, 1BJV, 1BRA, 1BTY, 1C1R, 1C5P, 1C5Q, 1C5S, 1C5T, 1CE5, 1EB2, 1SFI, 1F0T, 1F0U, 1G36, 1G3B, 1G3C, 1G3D, 1G3E, 1GBT, 1GGD, 1GHZ, 1GI1, 1GI4, 1GI6, 1GJ6, 1H4W, 1J14, 1J15, 1J16, 1J17, 1JRS, 1K1I, 1K1J, 1K1L, 1K1M, 1K1N, 1K1O, 1K1P, 1K2I, 1LQE, 1NC6, 1O2H, 1O2J, 1O2K, 1O2N, 1O2O, 1O2P, 1O2Q, 1O2R, 1O2S, 1O2T, 1O2U, 1O2V, 1O2W, 1O2X, 1O2Y, 1O2Z, 1O30, 1O32, 1O33, 1O34, 1O35, 1O36, 1O37, 1O38, 1O39, 1O3B, 1O3C, 1O3D, 1O3E, 1O3F, 1O3G, 1O3H, 1O3I, 1O3J, 1O3K, 1O3L, 1OSS, 1OXG, 1OYQ, 1PPC, 1PPH, 1QB1, 1QB6, 1QB9, 1QBN, 1QBO, 1QCP, 1QL7, 1QL9, 1RXP, 1TNG, 1TNH, 1TNI, 1TNJ, 1TNK, 1TNL, 1TPS, 1TX7, 1TYN, 1UTJ, 1UTL, 1UTM, 1UTN, 1UTO, 1UTP, 1V2J, 1V2K, 1V2L, 1V2M, 1V2N, 1V2O, 1V2P, 1V2Q, 1V2R, 1V2S, 1V2T, 1V2U, 1V2V, 1V2W, 1VGC, 1XUG, 1Y3V, 1Y3W, 1Y3X, 1Y3Y, 1YP9, 1YYY, 1ZZZ, 2A31, 2BZA, 2FX6, 2J9N, 2P8O, 2TPI, 2VGC, 2ZDK, 2ZDL, 2ZDM, 2ZDN, 2ZFS, 2ZFT, 2ZHD, 2ZQ1, 2ZQ2, 3AAS, 3AAU, 3AAV, 3ATI, 3ATK, 3ATL, 3ATM, 3GY2, 3GY3, 3GY4, 3GY7, 3LJJ, 3LJO, 3M35, 3NK8, 3NKK, 3RXA, 3RXB, 3RXD, 3RXE, 3RXF, 3RXG, 3RXH, 3RXI, 3RXJ, 3RXK, 3RXL, 3RXM, 3RXO, 3RXP, 3VGC, 4AB8, 4AB9, 4ABA, 4ABB, 4ABD, 4ABE, 4ABF, 4ABG, 4ABH, 4ABI, 4ABJ, 4GVU, 4HGC, 4VGC, 6CHA, 6GCH, 7GCH

Checkpoint kinase 1 complexes (N = 61)

1NVQ, 1NVR, 1NVS, 2BR1, 2BRB, 2BRG, 2BRH, 2BRM, 2BRN, 2BRO, 2C3J, 2C3L, 2CGU, 2CGW, 2CGX, 2E9N, 2E9O, 2E9U, 2E9V, 2HOG, 2HXL, 2HXQ, 2HY0, 2QHM, 2QHN, 2R0U, 2WMR, 2WMV, 2WMW, 2WMX, 2X8D, 2X8E, 2X8I, 2XEZ, 2XF0, 2YDI, 2YDJ, 2YDK, 2YER, 2YEX, 2YM3, 2YM4, 2YM5, 2YM6, 2YM7, 2YM8, 3F9N, 3JVR, 3JVS, 3NLB, 3OT3, 3OT8, 3PA3, 3PA4, 3PA5, 3TKH, 3TKI, 3U9N, 4HYH, 4HYI, 4JIK

Table S2. Basic information of the HIV-1 protease data set used in the molecular docking test

Ligand ID [a]	Inhibition constant (K_i, nM) [a]	logK_a	Computed binding scores (logK_a)		PDB ID of the reference complex selected by KGS2
			ChemPLP	ChemPLP + KGS2	
BDBM13925	0.0045	11.35	7.16	12.75	4KB9
BDBM8125	0.014	10.85	7.01	12.69	4KB9
BDBM9236	0.014	10.85	6.79	12.43	4KB9
BDBM9269	2.5	8.60	6.79	12.47	4KB9
BDBM9278	2.2	8.66	6.74	12.42	4KB9
BDBM9277	1.2	8.92	6.91	12.54	4KB9
BDBM9272	1.5	8.82	6.85	12.42	4KB9
BDBM9273	1.5	8.82	6.85	12.52	4KB9
BDBM9270	1.2	8.92	6.90	12.57	4KB9
BDBM9271	1.4	8.85	6.57	12.15	4KB9
BDBM13924	0.14	9.85	7.09	12.66	4KB9
BDBM13926	5.3	8.27	7.01	12.38	4KB9

[a]: Ligand ID and binding data were taken from BindingDB.

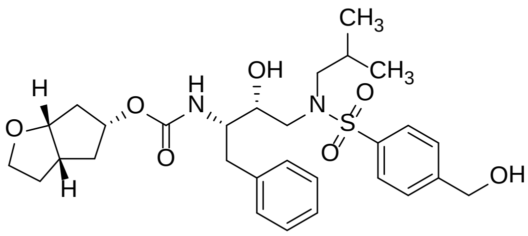

BDBM13925

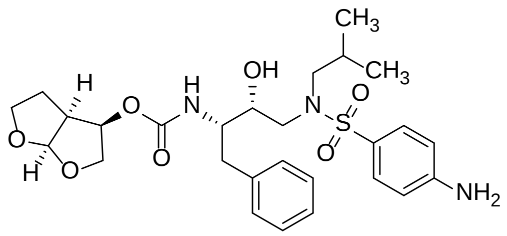

BDBM8125

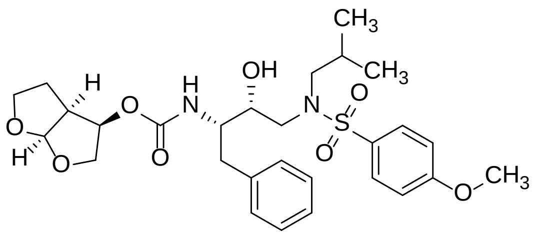

BDBM9236

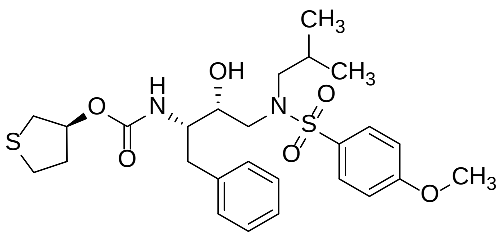

BDBM9269

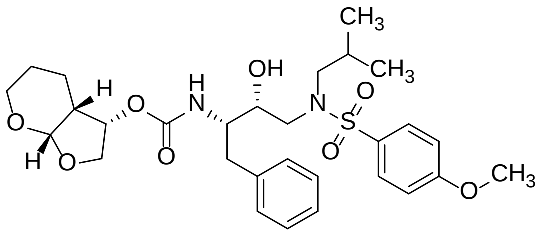

BDBM9278

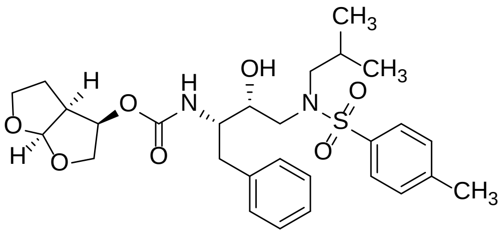

BDBM9277

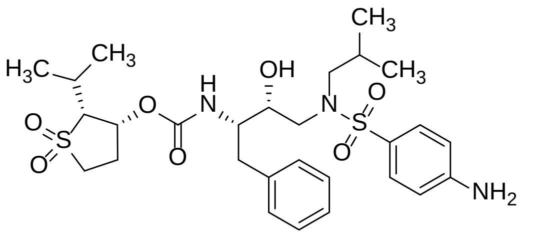

BDBM9272

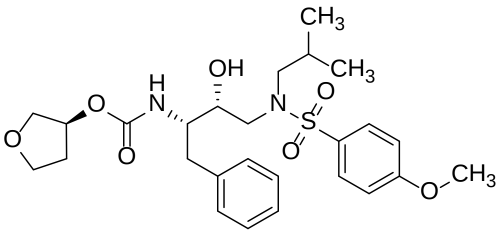

BDBM9273

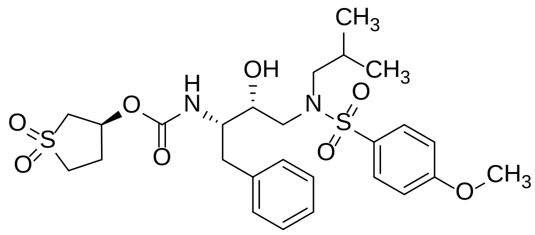

BDBM9270

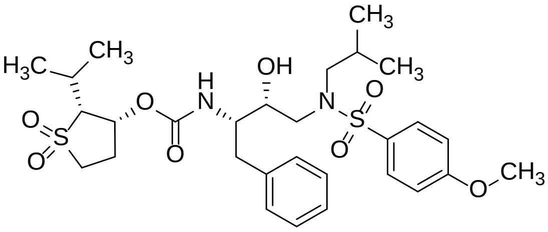

BDBM9271

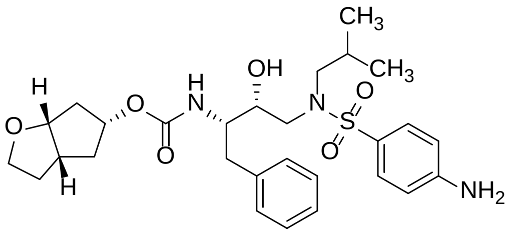

BDBM13924

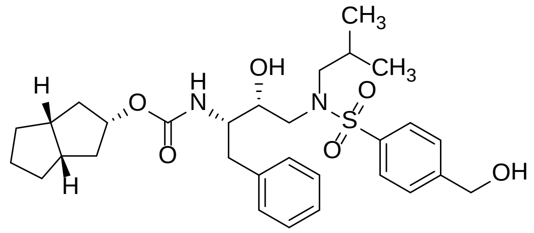

BDBM13926

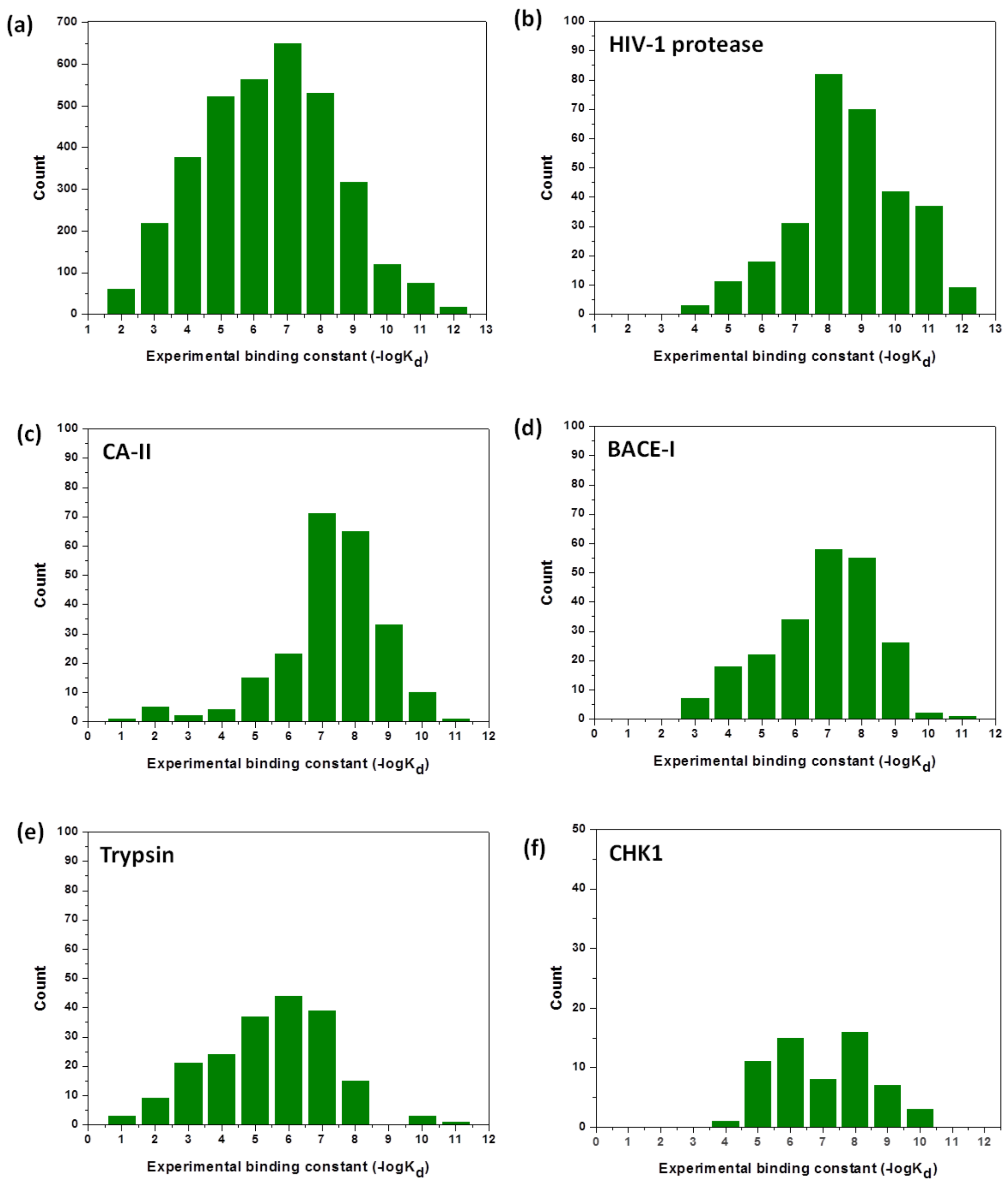

Figure S1. Distributions of the experimental binding constants of the protein-ligand complexes in (a) the PDBbind refined set version 2014; (b) The HIV-1 protease test set; (c) The carbonic anhydrase 2 test set; (d) The beta-secretase test set; (e) The beta-trypsin test set. (f) The checkpoint kinase 1 test set.

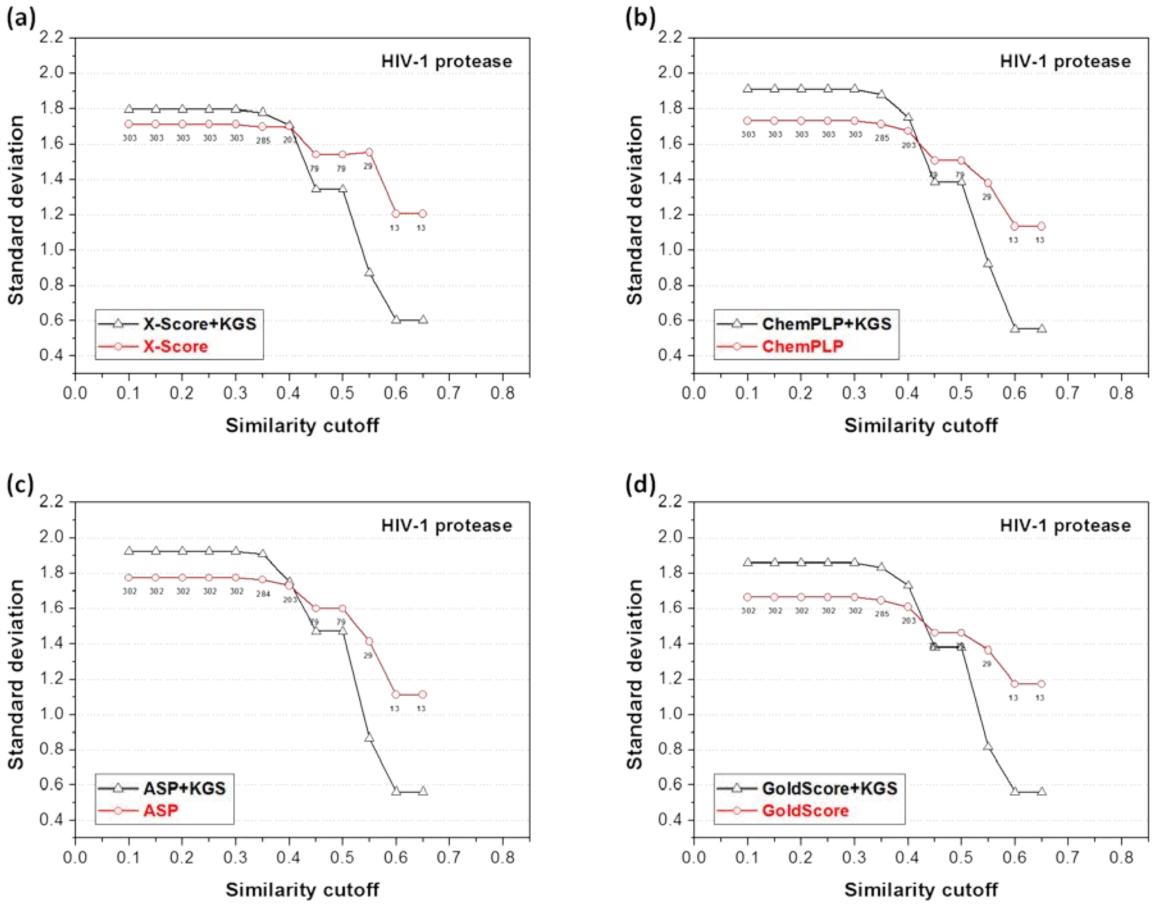

Figure S2. Results produced by (a) X-Score, (b) ChemPLP, (c) ASP, and (d) GoldScore in couple with KGS on the HIV-1 protease test set. All annotations in this figure are similar to those used in Figure 5 in the main text.

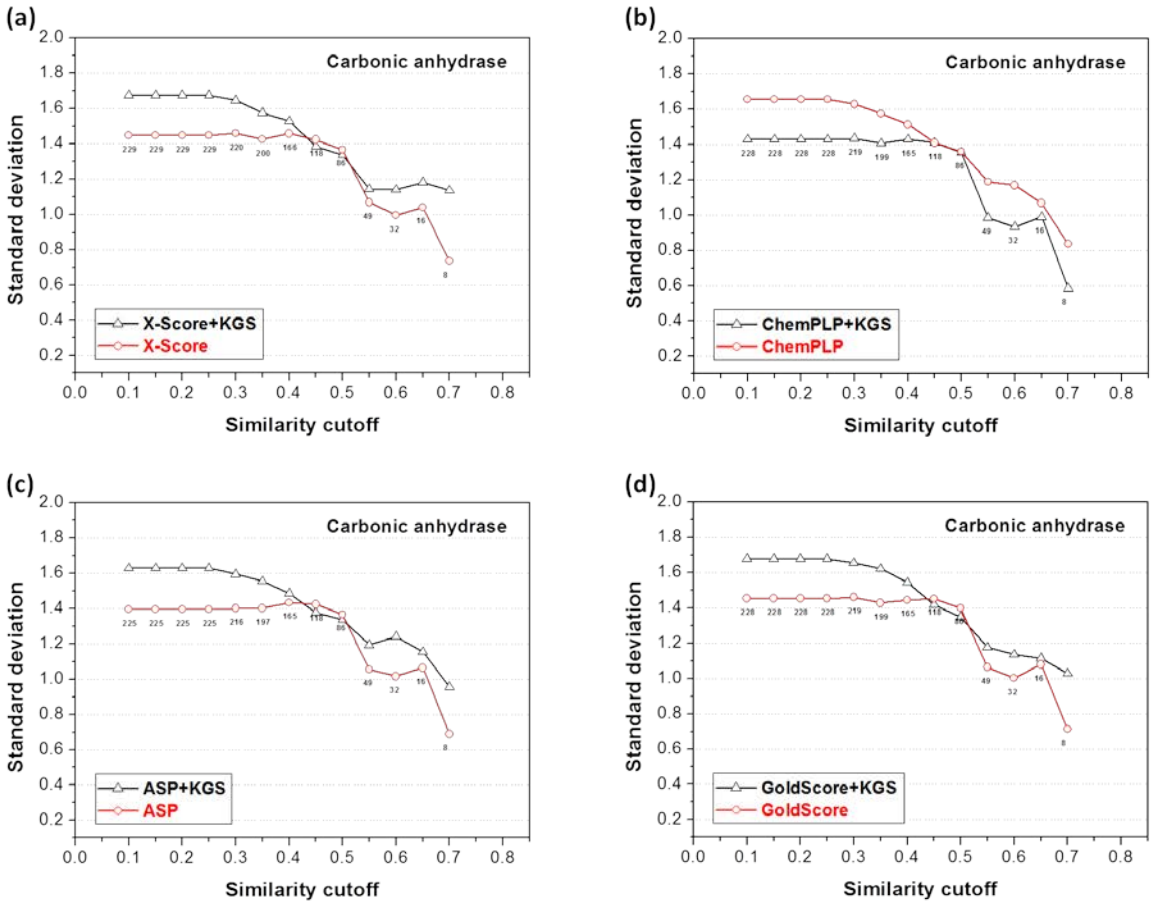

Figure S3. Results produced by (a) X-Score, (b) ChemPLP, (c) ASP, and (d) GoldScore in couple with KGS on the carbonic anhydrase 2 test set. All annotations in this figure are similar to those used in Figure 5 in the main text.

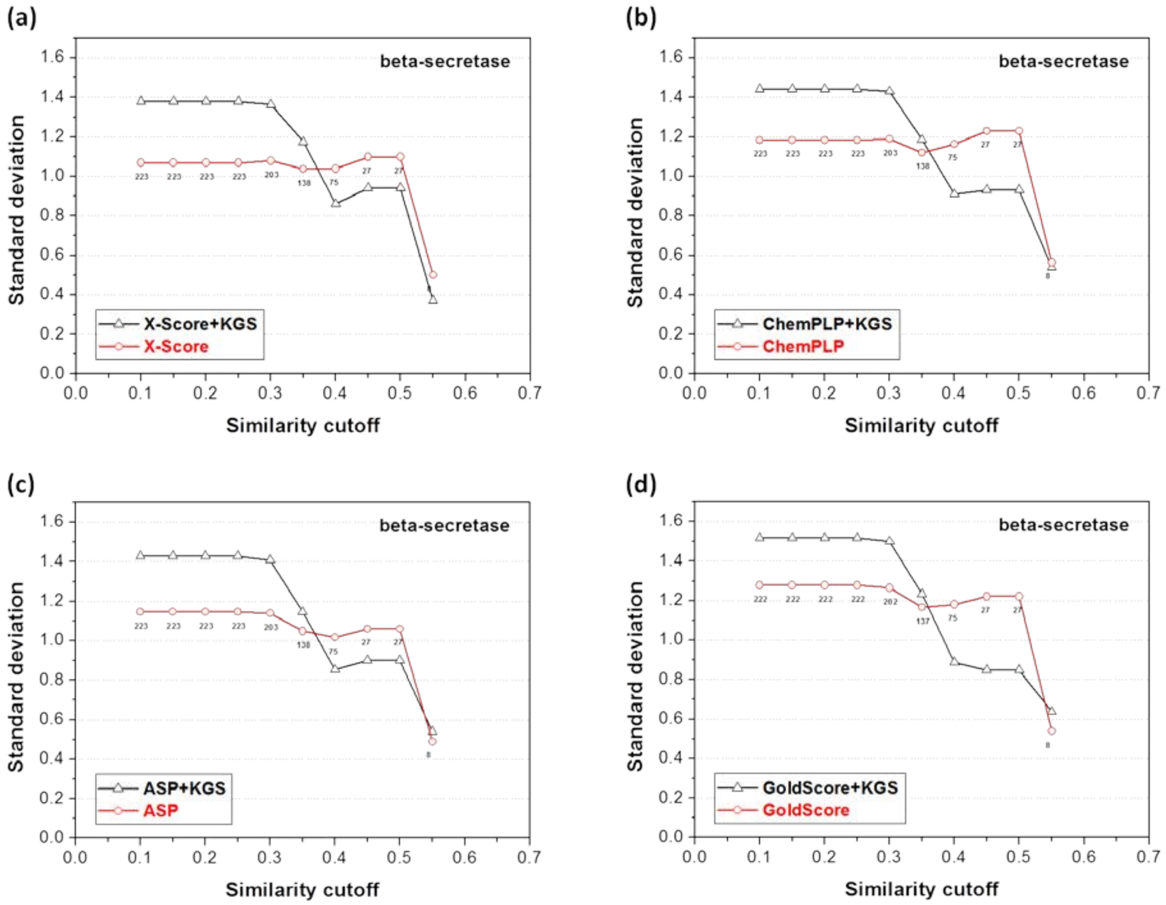

Figure S4. Results produced by (a) X-Score, (b) ChemPLP, (c) ASP, and (d) GoldScore in couple with KGS on the beta-secretase 1 test set. All annotations in this figure are similar to those used in Figure 5 in the main text.

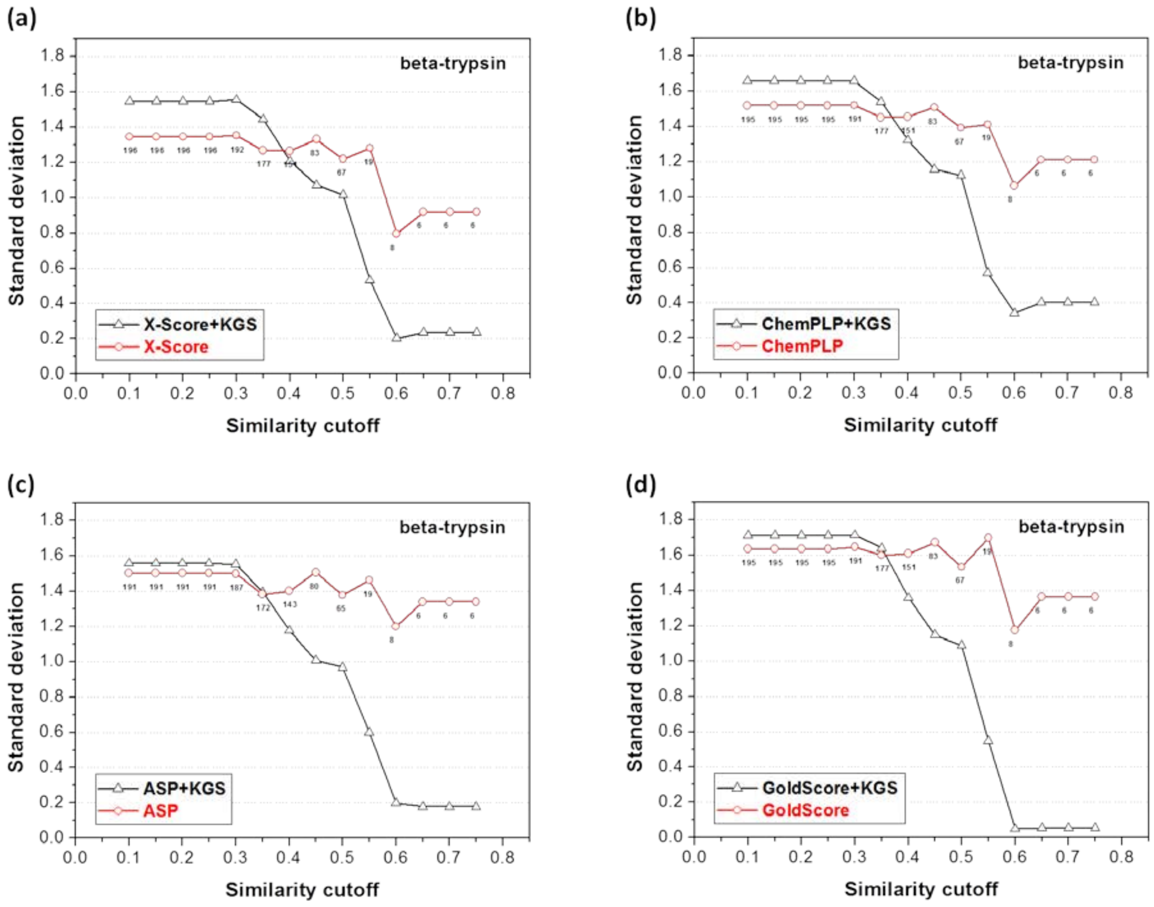

Figure S5. Results produced by (a) X-Score, (b) ChemPLP, (c) ASP, and (d) GoldScore in couple with KGS on the beta-trypsin test set. All annotations in this figure are similar to those used in Figure 5 in the main text.

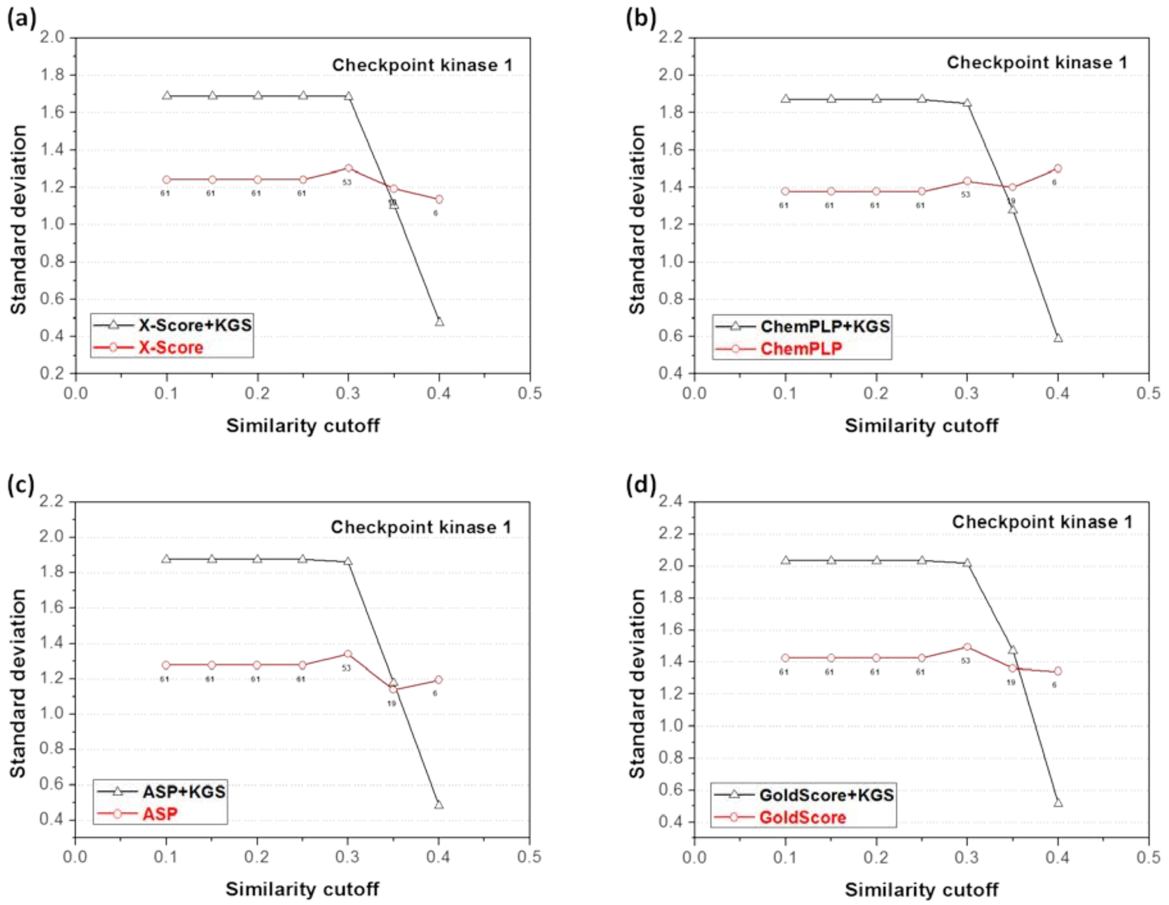

Figure S6. Results produced by (a) X-Score, (b) ChemPLP, (c) ASP, and (d) GoldScore in couple with KGS on the checkpoint kinase 1 test set. All annotations in this figure are similar to those used in Figure 5 in the main text.

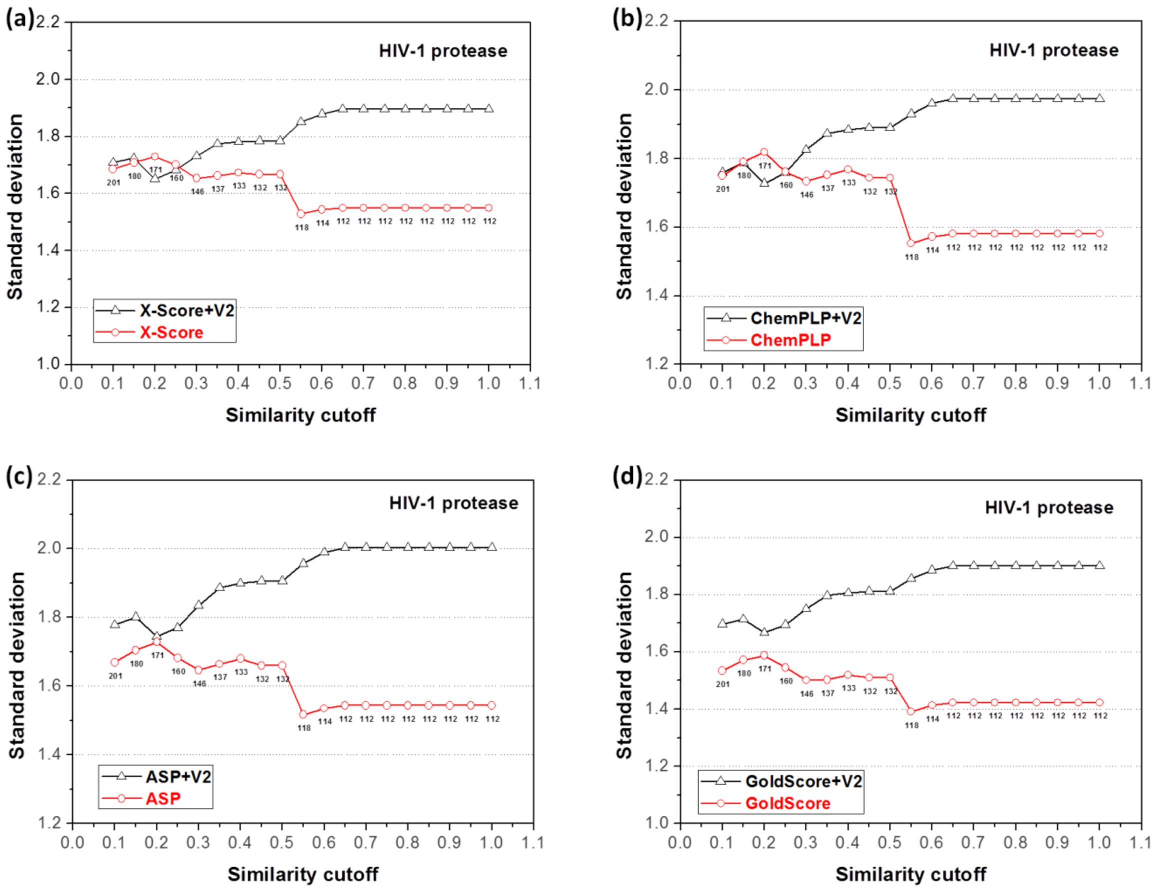

Figure S7. Results produced by (a) X-Score, (b) ChemPLP, (c) ASP, and (d) GoldScore in couple with Variation Model 2 on the HIV-1 protease test set. All annotations in this figure are similar to those used in Figure 5 in the main text.

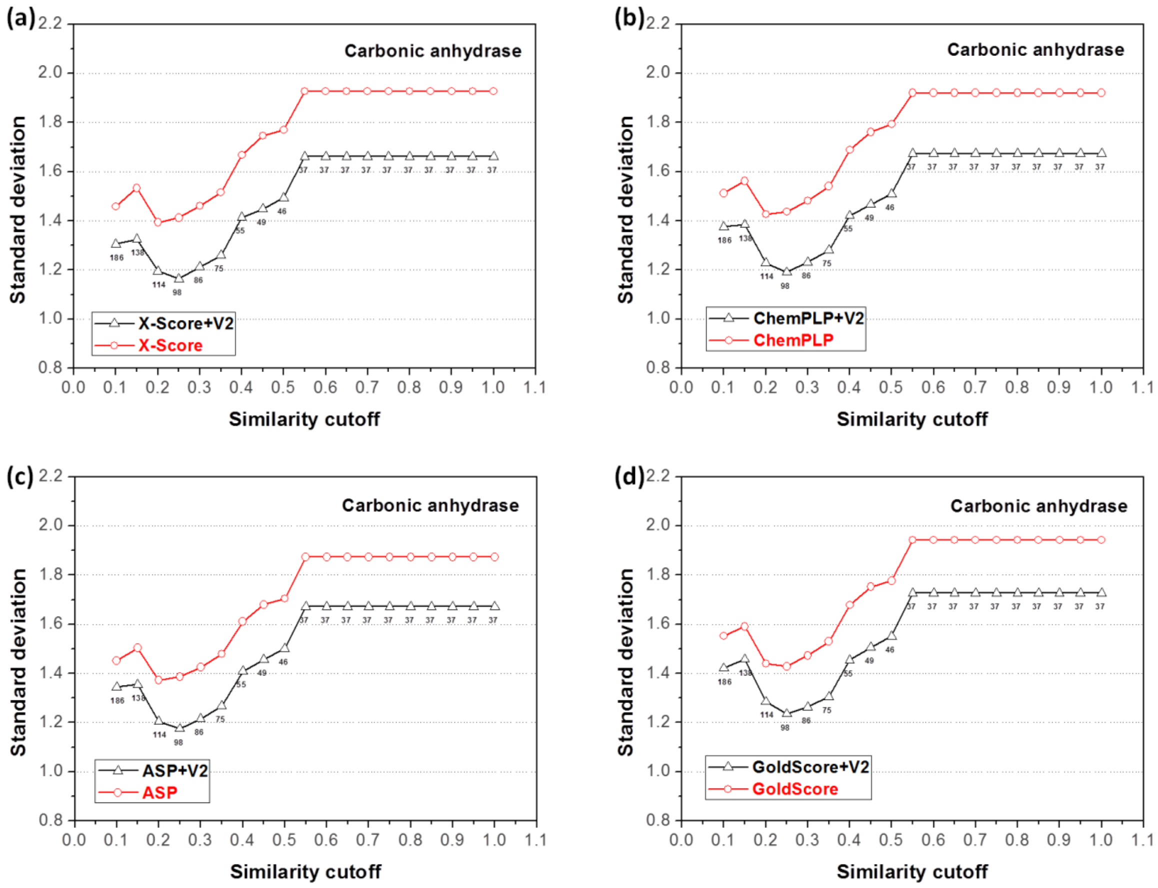

Figure S8. Results produced by (a) X-Score, (b) ChemPLP, (c) ASP, and (d) GoldScore in couple with Variation Model 2 on the carbonic anhydrase 2 test set. All annotations in this figure are similar to those used in Figure 5 in the main text.

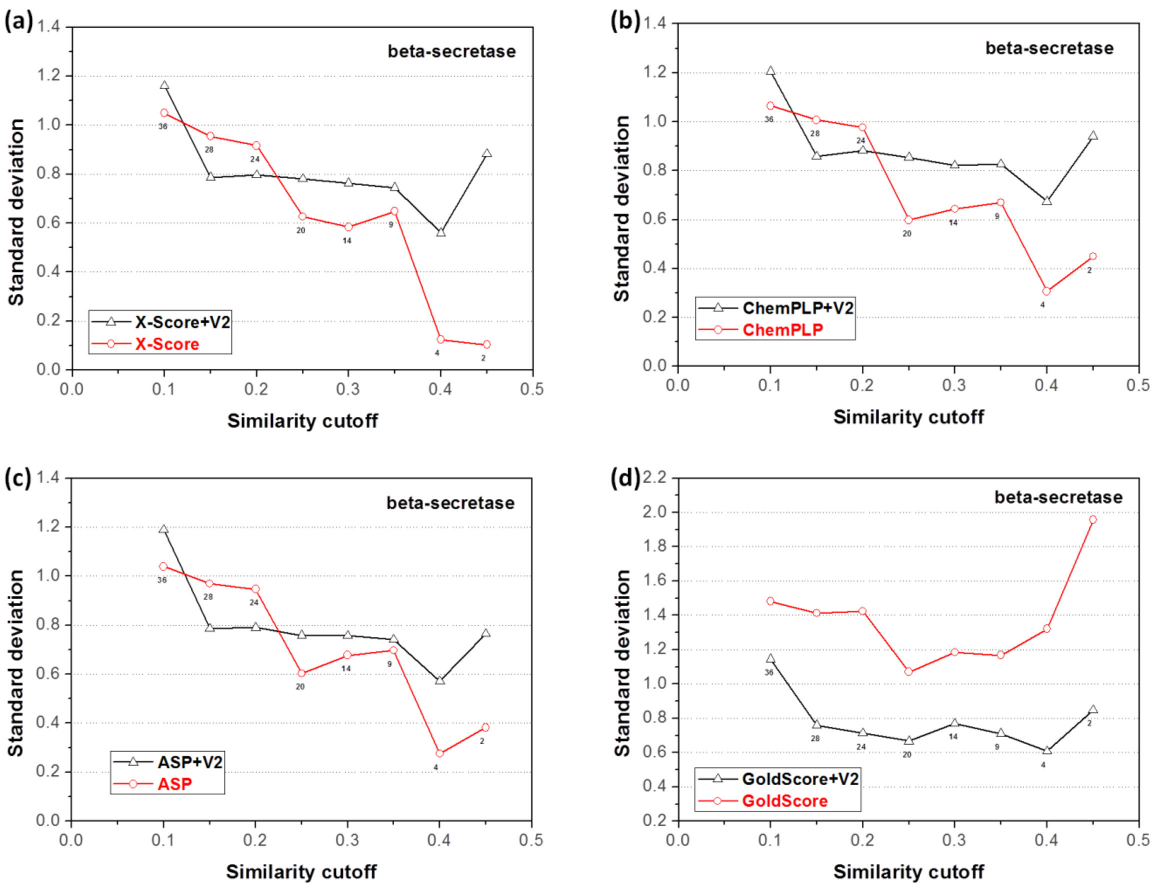

Figure S9. Results produced by (a) X-Score, (b) ChemPLP, (c) ASP, and (d) GoldScore in couple with Variation Model 2 on the beta-secretase 1 test set. All annotations in this figure are similar to those used in Figure 5 in the main text.

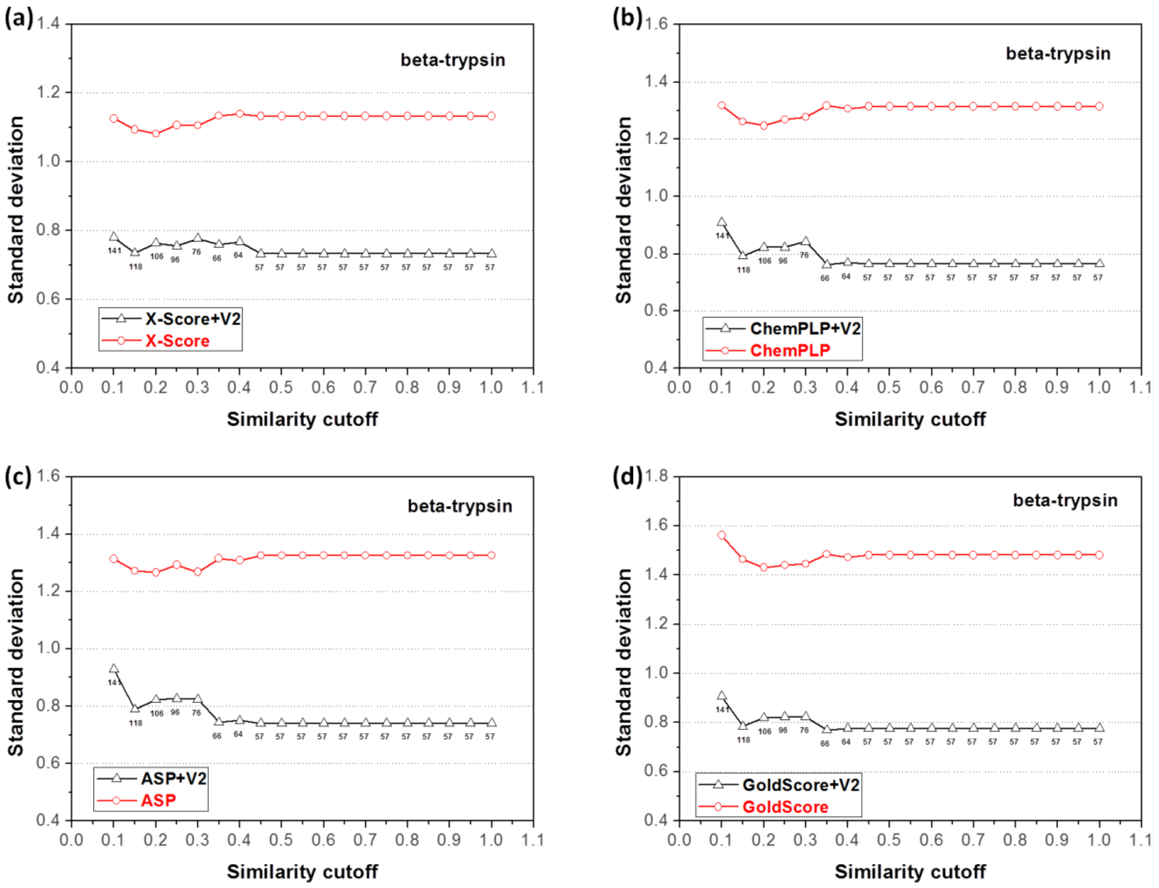

Figure S10. Results produced by (a) X-Score, (b) ChemPLP, (c) ASP, and (d) GoldScore in couple with Variation Model 2 on the beta-trypsin test set. All annotations in this figure are similar to those used in Figure 5 in the main text.

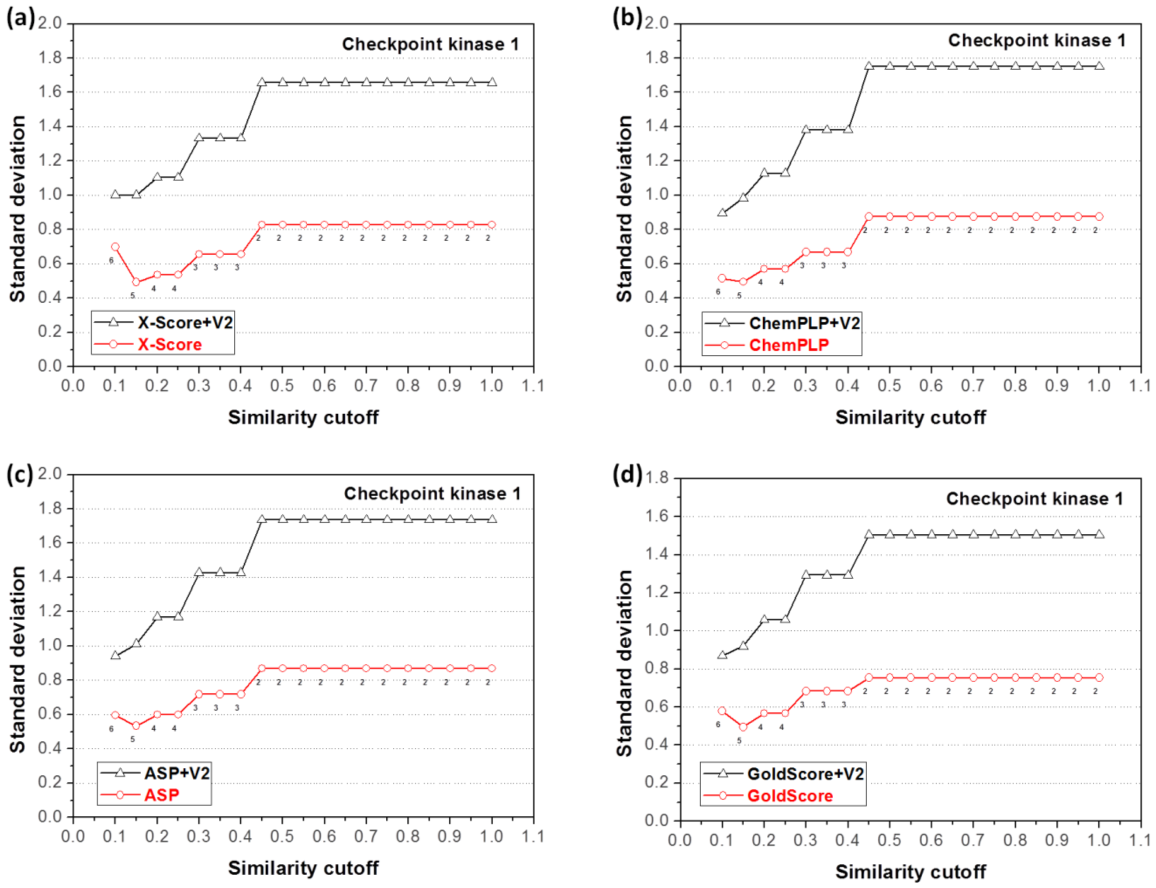

Figure S11. Results produced by (a) X-Score, (b) ChemPLP, (c) ASP, and (d) GoldScore in couple with Variation Model 2 on the checkpoint kinase 1 test set. All annotations in this figure are similar to those used in Figure 5 in the main text.

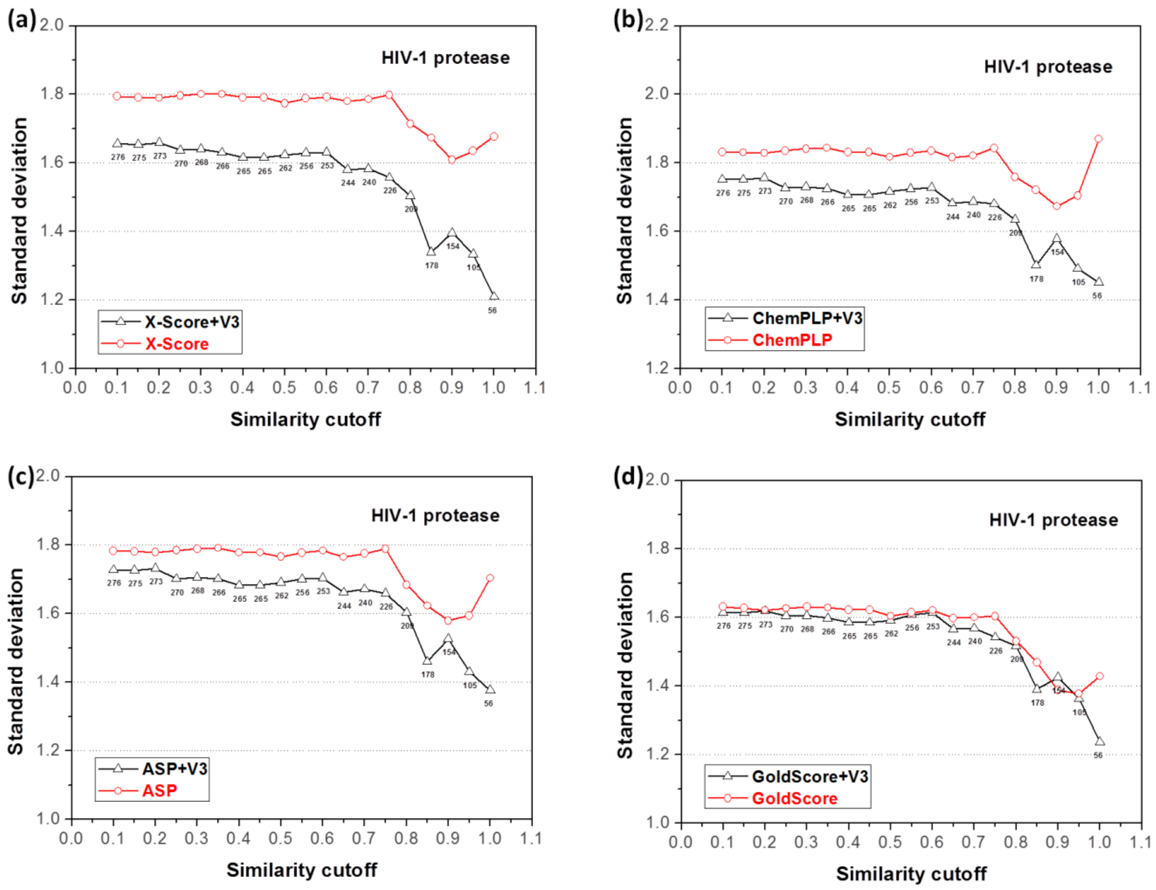

Figure S12. Results produced by (a) X-Score, (b) ChemPLP, (c) ASP, and (d) GoldScore in couple with Variation Model 3 on the HIV-1 protease test set. All annotations in this figure are similar to those used in Figure 5 in the main text.

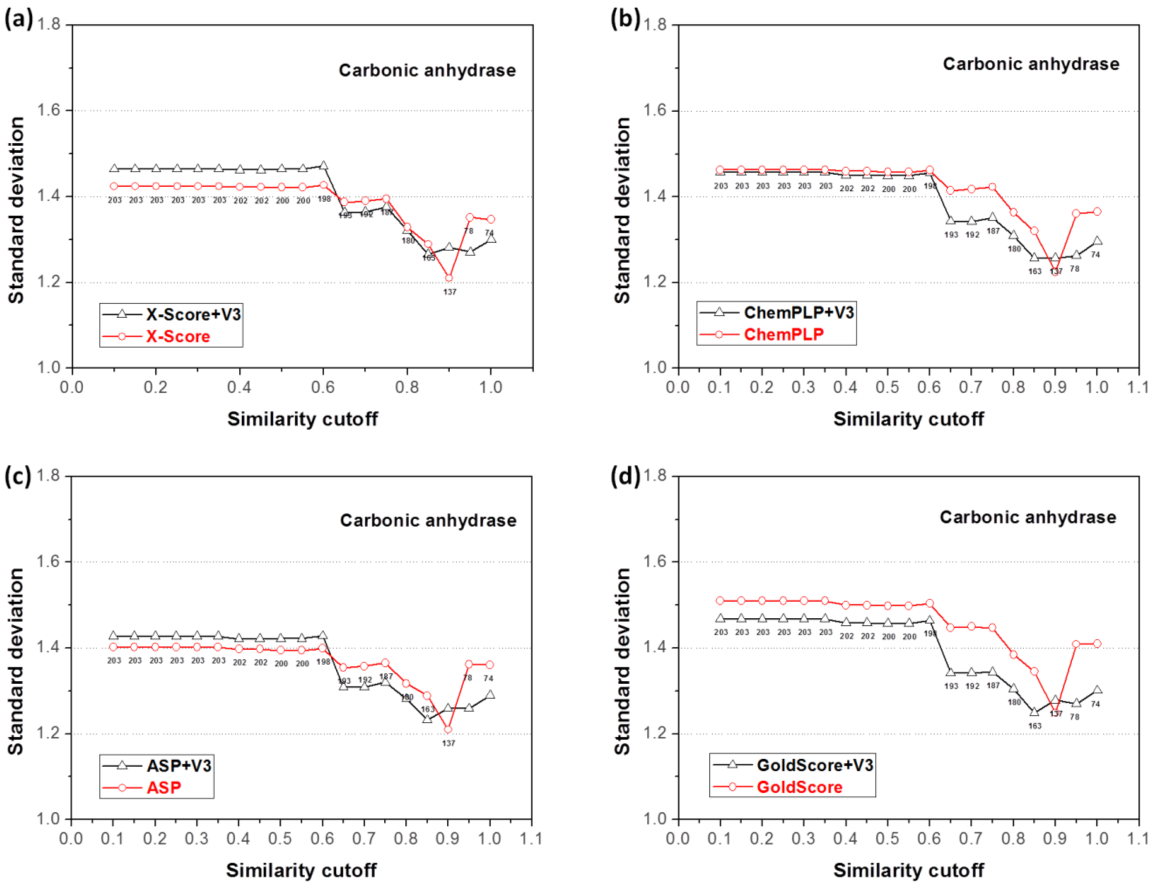

Figure S13. Results produced by (a) X-Score, (b) ChemPLP, (c) ASP, and (d) GoldScore in couple with Variation Model 3 on the carbonic anhydrase 2 test set. All annotations in this figure are similar to those used in Figure 5 in the main text.

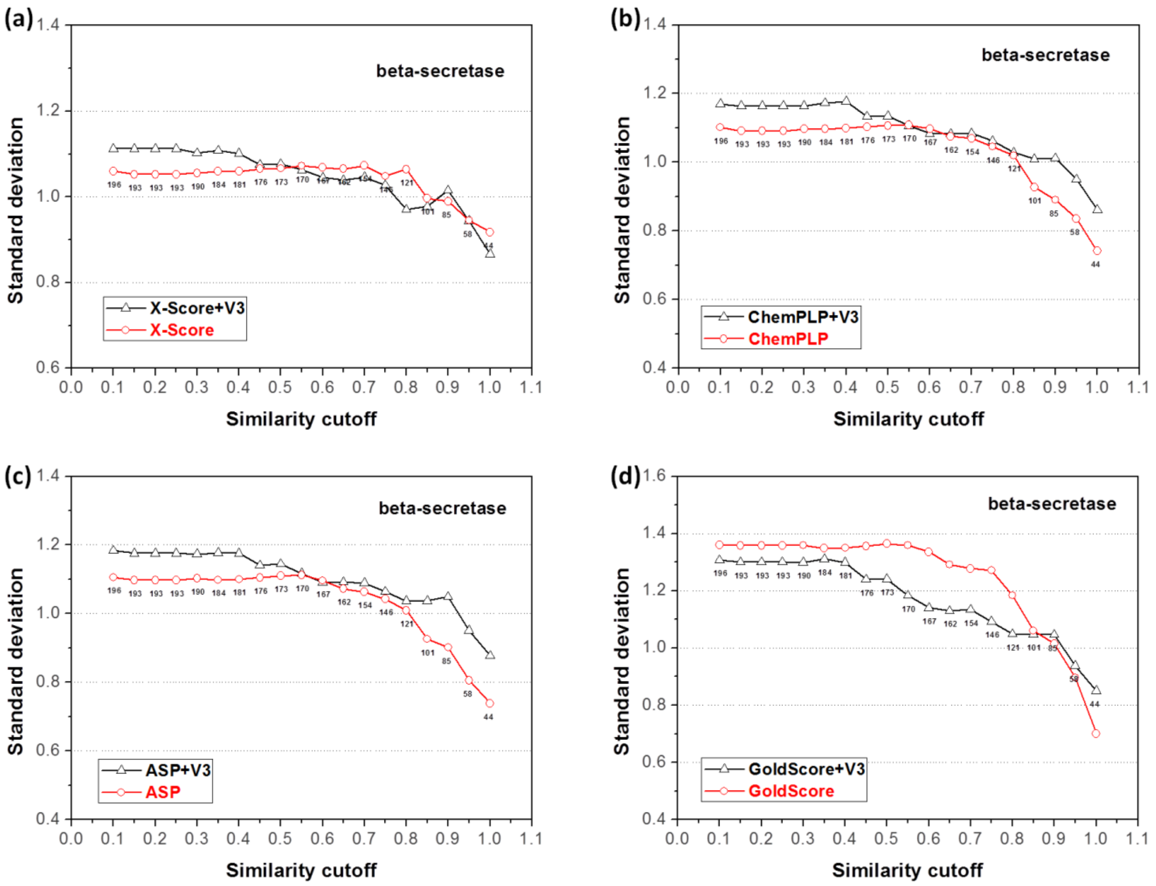

Figure S14. Results produced by (a) X-Score, (b) ChemPLP, (c) ASP, and (d) GoldScore in couple with Variation Model 3 on the beta-secretase 1 test set. All annotations in this figure are similar to those used in Figure 5 in the main text.

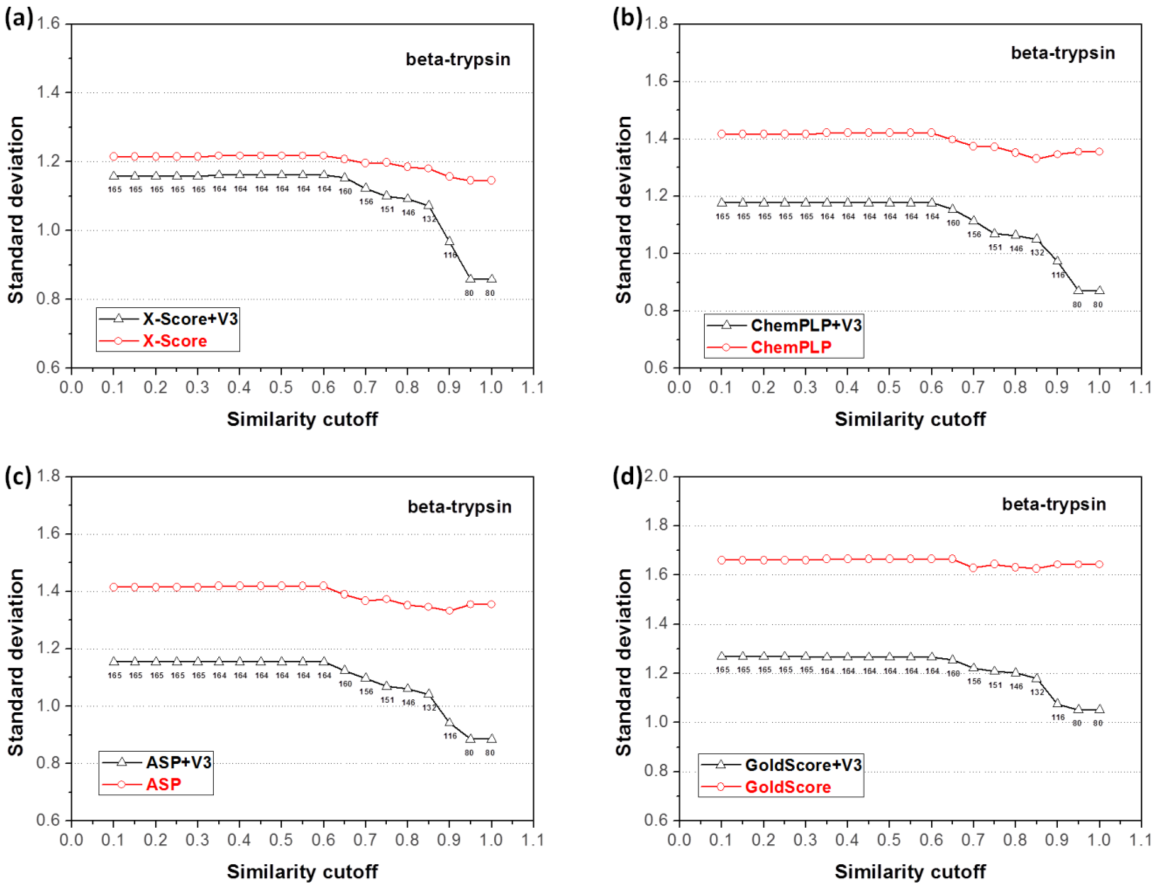

Figure S15. Results produced by (a) X-Score, (b) ChemPLP, (c) ASP, and (d) GoldScore in couple with Variation Model 3 on the beta-trypsin test set. All annotations in this figure are similar to those used in Figure 5 in the main text.

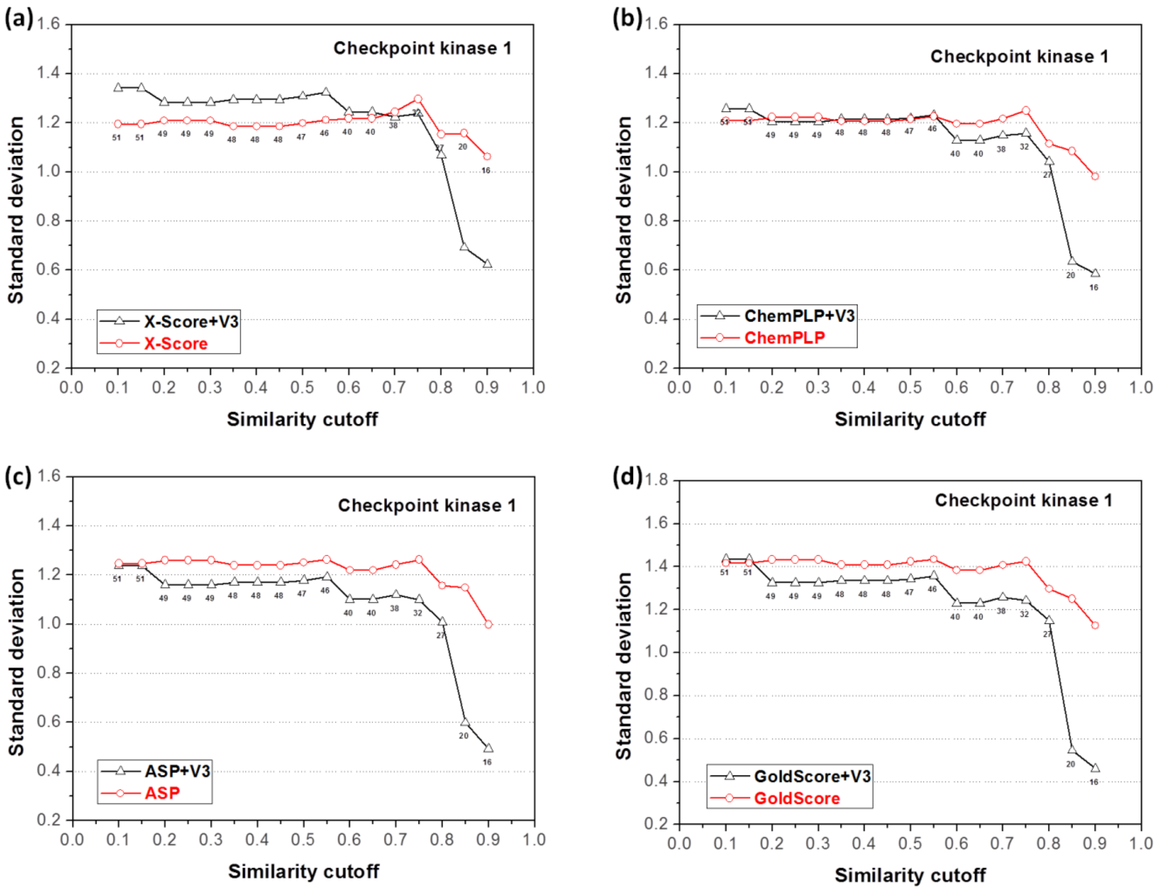

Figure S16. Results produced by (a) X-Score, (b) ChemPLP, (c) ASP, and (d) GoldScore in couple with Variation Model 3 on the checkpoint kinase 1 test set. All annotations in this figure are similar to those used in Figure 5 in the main text.
